# Pro-invasive properties of Snail1 are regulated by sumoylation in response to TGFβ stimulation in cancer

**DOI:** 10.18632/oncotarget.20097

**Published:** 2017-08-09

**Authors:** Shyam Kumar Gudey, Reshma Sundar, Carl-Henrik Heldin, Anders Bergh, Marene Landström

**Affiliations:** ^1^ Department of Medical Biosciences, Umeå University, Umeå, Sweden; ^2^ Ludwig Institute for Cancer Research, Science for Life Laboratory, Uppsala University, Uppsala, Sweden

**Keywords:** signal transduction, tumor biology, Snail1, sumoylation, prostate cancer

## Abstract

Transforming growth factor β (TGFβ) is a key regulator of epithelial-to-mesenchymal transition (EMT) during embryogenesis and in tumors. The effect of TGFβ, on ΕΜΤ, is conveyed by induction of the pro-invasive transcription factor Snail1. In this study, we report that TGFβ stimulates Snail1 sumoylation in aggressive prostate, breast and lung cancer cells. Sumoylation of Snail1 lysine residue 234 confers its transcriptional activity, inducing the expression of classical EMT genes, as well as TGFβ receptor I (TβRI) and the transcriptional repressor Hes1. Mutation of Snail1 lysine residue 234 to arginine (K234R) abolished sumoylation of Snail1, as well as its migratory and invasive properties in human prostate cancer cells. An increased immunohistochemical expression of Snail1, Sumo1, TβRI, Hes1, and c-Jun was observed in aggressive prostate cancer tissues, consistent with their functional roles in tumorigenesis.

## INTRODUCTION

Transforming growth factor beta (TGFβ) is a versatile cytokine implicated in crucial cellular processes such as embryogenesis, differentiation, proliferation, apoptosis, and tissue repair [1, 2]. TGFβ was discovered in 1980 and was originally given its name because of its ability to promote anchorange-independent growth of rat fibroblasts. In contrast, TGFβ was later found to inhibit proliferation of epithelial cells and maintain their homeostasis; Thus TGFβ has both pro-tumorigenic and tumor suppressive effects [1, 3–5]. TGFβ exhibits its growth-suppressive effects at initial stages by limiting cell proliferation and cell migration, and inducing apoptosis in normal epithelial cells [6, 7]. At later stages, however, TGFβ promotes tumor growth by evading these inhibitory signals and instead triggering other cellular processes such as the epithelial-to-mesenchymal transition (EMT), enabling cells to become motile and traverse to distant organs and metastasize [6, 8].

TGFβ signals by forming a heterotetrameric complex of two types of serine/threonine receptor kinases, the TGFβ receptors (TβRs) II and I [9]. Ligand binding to the TβR complex activates the TβRI kinase, which phosphorylates the receptor-associated Smads (R-Smads) 2 and 3, at their extreme C-terminal motifs, allowing them to form complexes with the co-Smad, Smad 4 [10–12]. The trimeric Smad complexes then translocate to the nucleus and promote TGFβ-induced transcriptional responses by binding to the Smad Binding Elements (SBEs) composed of the sequence CAGACA on the DNA [13, 14].

Intriguingly, TGFβ also drives transcriptional responses through other signaling pathways, referred to as non-Smad signaling, e.g. via ERK, JNK, and p38 mitogen activated protein kinases (MAPK) [7, 15, 16]. In this context, our group has identified that the E3 ligase tumor necrosis factor receptor (TNFR) associated factor 6 (TRAF6) plays a pivotal role in non-Smad signaling pathways [17]. TGFβ induced oligomerization of TβRII-TβRI complex promotes auto-ubiquitination of TRAF6 and subsequent activation of the TAK1-MKK3/6-p38-MAPK pathway [17–19]. TRAF6 also promotes cleavage of TβRI by activating the proteolytic proteases TACE (TNF-α converting enzyme), known as ADAM17, and presenilin1, in the ecto- and transmembrane regions, respectively [20, 21]. The cleaved TβRI-intracellular domain (TβRI-ICD) translocates to the nucleus where it binds to the transcription regulator p300 and activates pro-invasive genes, such as *Snail1* and *MMP2* [22].

One of the hallmarks of the EMT process is the repression of epithelial markers, such as E-cadherin and occludin, and upregulation of mesenchymal markers such as vimentin, N-cadherin, and fibronectin1 [23]. Key transcriptional regulators, such as *Snail1, Twist1, Zeb1*, and *Slug* play pivotal roles in this process [24].

TGFβ is well known to drive the EMT process by inducing the expression of Snail1 and other transcription factors, thereby regulating the expression of crucial mesenchymal markers [25] [26]. Interaction between signaling components of the TGFβ family and Snail1 has been reported. Smad3 and Smad4 form a complex with Snail1, driving EMT in breast carcinomas [27]. The high mobility group A2 (HMGA2) protein directly binds to the Snail1 promoter and acts as a transcriptional regulator of Snail1 expression [28]. Snail1 has also been reported to bind to its own promoter and regulate its own expression [29]. Previously, we have reported that the AP-1 transcription factor *c-Jun* binds to a distal region of the Snail1 promoter and thereby promotes invasion of prostate cancer cells [30, 31].

Moreover, various post-translational modifications regulate the stability and activity of Snail1 protein expression. For instance, Snail1 has been reported to undergo polyubiquitination in the nucleus by F-box protein FBXL5, thereby hampering Snail1 ability to bind to DNA [32]. Sumoylation i.e. the conjugation of a small ubiquitin-like modifier (SUMO) to the target substrate, regulates protein stability, nucleo-cytoplasmic shuttling, active gene transcription, chromosome organization and DNA repair [33]. Conjugation of sumo moieties to the target lysine residue occurs either by monoSUMOylation or by attachment of SUMO chains (polySUMOylation) [34, 35].

Although it has been reported that there is cross-talk between Smads and Snail1, it is still unclear which downstream targets of Snail1 promote EMT in TGFβ stimulated cells. In this study, we explored the downstream regulators of Snail1, and found that Snail1 regulates both mRNA and protein expression of TβRI and the transcriptional repressor Hes1. Moreover, we observe that knockdown of the TβRI decreases the expression of Hes1 and EMT-related genes. We also found that TGFβ promotes sumoylation of Snail1. Moreover, mutagenesis of Snail1 lysine residue 234 to arginine (K234R) abolished sumoylation of Snail1, its transcriptional activity, and migration and invasion of prostate cancer cells.

## RESULTS

### Snail1 regulates TβRI expression

**Figure 1:**
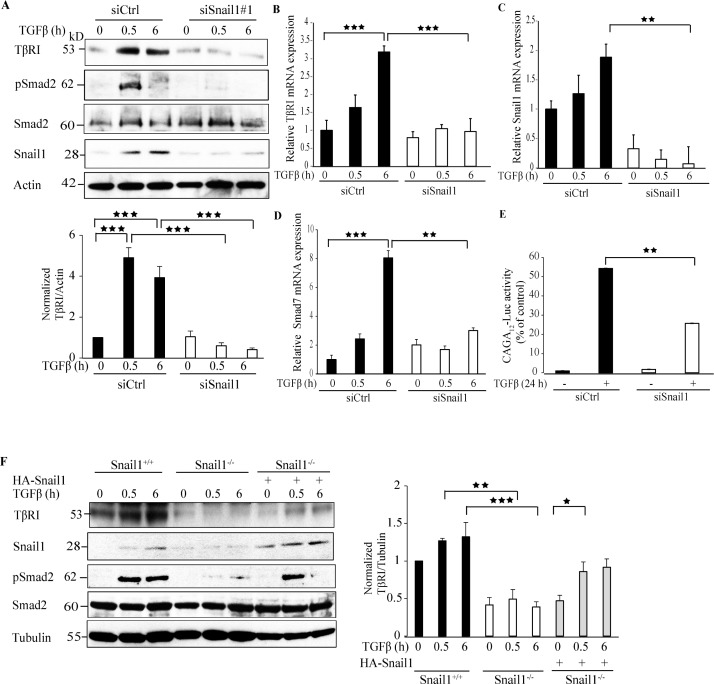
Snail1 regulates TβRI expression **A**. PC-3U cells were transiently transfected with control (Ctrl) or Snail1-specific siRNA #1 and treated with TGFβ (10 ng/ml) for the indicated time periods. Cell lysates were prepared and immunoblots were probed for TβRI (TβRI-FL), pSmad2, and Smad2, Snail1, and β-actin, which served as control for equal loading of proteins (*n* = 5 independent experiments). **B**.-**D**. qRT-PCR analysis of PC-3U cells transiently transfected with control (Ctrl) or Snail1-specific siRNA #1 and treated with TGFβ (10 ng/ml) as indicated. RNA was extracted and cDNA was prepared and used for qRT-PCR analysis of mRNA expression of TβRI, Snail1, and Smad7 respectively (*n* = 4 independent experiments). **E**. PC-3U cells were transiently transfected with control (Ctrl) or Snail1-specific siRNA #1 followed by transfection of CAGA_12_-Luc reporter. Later, cells were starved and treated without or with TGFβ (10 ng/ml) for 24h and luciferase activity was measured. (*n* = 5 independent experiments). **F**. Snail1^+/+^, Snail1^−/−^ MEFs, and Snail1^−/−^ MEFs transiently transfected with HA-Snail1 were serum starved and treated with TGFβ for the indicated time periods. Cell lysates were prepared and immunoblots were probed for TβRI (TβRI-FL), Snail1, pSmad2, and Smad2, β-tubulin (*n* = 4 independent experiments). Bar graphs show the means ± SEM; ^*^P < 0.05, ^**^P < 0.005, ^***^P < 0.0005. Differences in the means ± SEM between samples were analyzed with two-way ANOVA and Bonferroni correction for multiple comparisons.

To investigate whether Snail1 regulates the expression of TβRI, we silenced the endogenous expression of Snail1 by using siRNA or non-targeting control siRNA in PC-3U cells and probed with antibodies directed against TβRI. Downregulation of Snail1 expression by siRNA#1 or siRNA#2 decreased TβRI protein (TβRI) expression (Figure 1A and Supplementary Figure 1A). Moreover, silencing of Snail1 decreased TβRI mRNA expression, as determined by RT-PCR analysis (Figure 1B). Downregulation of Snail1 expression was confirmed at both the protein and mRNA levels (Figure 1A, 1C, and Supplementary Figure 1A). Because R-Smads (Smad2, Smad3) are the downstream regulators of TβRI, we investigated the effects of silencing of Snail1, on the phosphorylation of Smad2 by using phospho-specific antibodies; treatment with siSnail1 reduced the phosphorylation of Smad2 (Figure 1A). Moreover, the mRNA expression of the TGFβ target gene *Smad7* also decreased in the absence of Snail1 (Figure 1D). As downregulation of endogenous Snail1 expression decreased phosphorylation of Smad2, we investigated if the downregulation of TβRI by Snail1, inhibits Smads to bind to the Smad Binding Elements (SBEs) by performing Smad-specific promoter reporter assays by transfecting CAGA_12_-Luc reporters in siCtrl or siSnail1 treated PC-3U cells. Treatment with TGFβ for 24 h significantly enhanced CAGA_12_-Luc reporter activity in siCtrl treated cells compared to siSnail1 treated cells (Figure 1E). Next, we confirmed our findings that Snail1 downregulates TβRI protein expression in Snail1-deficient mouse embryo fibroblasts (MEFs). The TGFβ-enhanced expression of TβRI protein expression observed in Snail1^+/+^ MEFs was suppressed in Snail1^−/−^ MEFs (Figure 1F). Probing with phospho-Smad2 antiserum confirmed that TβRI stimulation caused phosphorylation of Smad2 only in Snail1^+/+^ MEFs, but not in Snail1^−/−^ MEFs (Figure 1F). Moreover, re-introduction of HA-Snail1 in Snail1^−/−^ MEFs partially rescued the expression of TβRI and phosphorylation of Smad2 (Figure 1F). By confocal imaging we observed that TGFβ stimulation of cells, enhanced the intensity and co-localization of TβRI and Snail1 in siCtrl treated cells but not in siSnail1 treated cells (Supplementary Figure 2A). We confirmed our findings in Snail1 deficient MEFs; whereas TGFβ treatment greatly enhanced the co-localisation of TβRI and Snail1 in Snail1^+/+^ MEFs, this was not seen in Snail1^−/−^ MEFs (Supplementary Figure 2B). These results suggest that Snail1 enhances both mRNA and protein expression of TβRI.

### Snail1 interacts with TβRI

**Figure 2:**
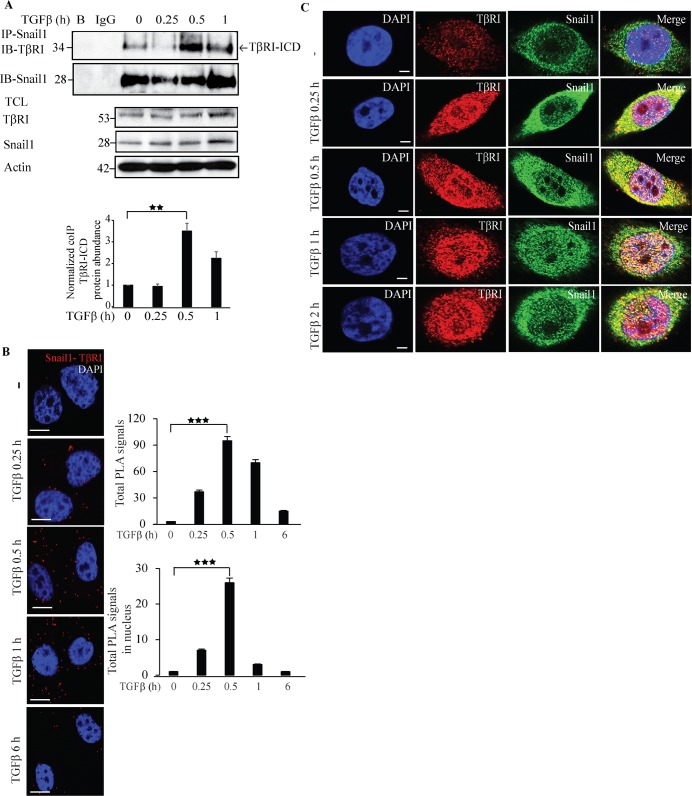
Snail1 interacts with TβRI **A**. PC-3U cells were treated with TGFβ for the indicated time periods. Cell lysates were prepared and immunoprecipitated with goat-Snail1 and immunoblotted with rabbit TβRI antibodies. Total cell lysates were probed for endogenous TβRI, Snail1, and β-actin (*n* = 4 independent experiments). **B**. PLA images of PC-3U cells were treated with TGFβ for the indicated time periods. Cells were fixed, permeabilised and incubated with anti-rabbit TβRI and anti-mouse Snail1 antibodies, followed by incubation with PLA probes. TβRI-Snail1 PLA complexes are visualized as red dots. Quantification of TβRI-Snail1 complexes was done with the aid of Blob finder software. (*n* = 4 independent experiments). **C**. Representative confocal imaging of PC-3U cells treated with TGFβ for the indicated time periods. Cells were fixed, permeabilised and incubated with anti-rabbit TβRI and anti-mouse Snail1 antibodies, followed by incubation with Alexa Fluor 555 (red) secondary anti-rabbit antibodies and Alexa Fluor 488 (green) secondary anti-mouse antibodies for visualization. Merge of two layers shows co-localization of the proteins. Cell nuclei stained with DAPI (*n* = 3 independent experiments). Scale bar, 20 μm. Bar graphs show the means ± SEM; ^**^P < 0.005, ^***^P < 0.0005. Differences in the means ± SEM between samples were analyzed with two-way ANOVA and Bonferroni correction for multiple comparisons.

Having observed that Snail1 regulates TβRI, we investigated if Snail1 forms a complex with TβRI in PC-3U cells. Interestingly, TGFβ induced a transient co-immunoprecipitation of endogenous Snail1 with TβRI-intracellular domain (TβRI-ICD) upon TGFβ stimulation (Figure 2A). By using proximity ligation assays (PLA), we confirmed that TGFβ treatment enhances Snail1-TβRI interaction in PC-3U cells (Figure 2B) and also in the highly invasive human breast cancer cell line MDA-MB-231 (Supplementary Figure 3). Moreover, confocal imaging showed that TGFβ stimulates co-localization between Snail1 and TβRI in the nuclear compartment in PC-3U cells (Figure 2C). These results suggest that TGFβ promotes interaction of TβRI and Snail1, in aggressive prostate and breast cancer cells.

To further investigate the interaction between Snail1 and TβRI, we ectopically co-expressed GFP-tagged TβRI and HA-Snail1 in PC-3U cells. As expected, TGFβ stimulated an interaction between GFP-TβRI and HA-Snail1 (Supplementary Figure 4A). Using confocal imaging, we observed that TGFβ stimulation promoted the co-localization of GFP-TβRI and HA-Snail1 in the nuclear compartment, although a small amount of proteins co-localized also in the cytoplasm (Supplementary Figure 4B). Next, we ectopically co-expressed Flag-tagged TβRI and HA-Snail1 in PC-3U cells; consistent with our previous finding, we observed TGFβ-dependent interaction between Flag-TβRI-ICD and HA-Snail1, further confirming that TGFβ stimulates complex formation between TβRI-ICD and Snail1 (Supplementary Figure 4C). Taken together, these results suggest that Snail1 forms a complex with TβRI-ICD, in the nucleus of TGFβ-treated prostate and breast cancer.

### Snail1 promotes EMT genes

Because Snail1 has been implicated as a major contributor to the EMT process, we performed qRT-PCR experiments to investigate if the mesenchymal genes are regulated by Snail1 in response to TGFβ. TGFβ triggered a Snail1-dependent upregulation of *Fibronectin1, N-cadherin,* and the Notch responsive gene *Jagged1* (Supplementary Figure 5A-5C). Moreover, Snail1 downregulation decreased the expression of other EMT regulators such as *Zeb1, Slug*, and *Twist1* (Supplementary Figure 5D-5F).

### Snail1 regulates Hes1 expression

**Figure 3:**
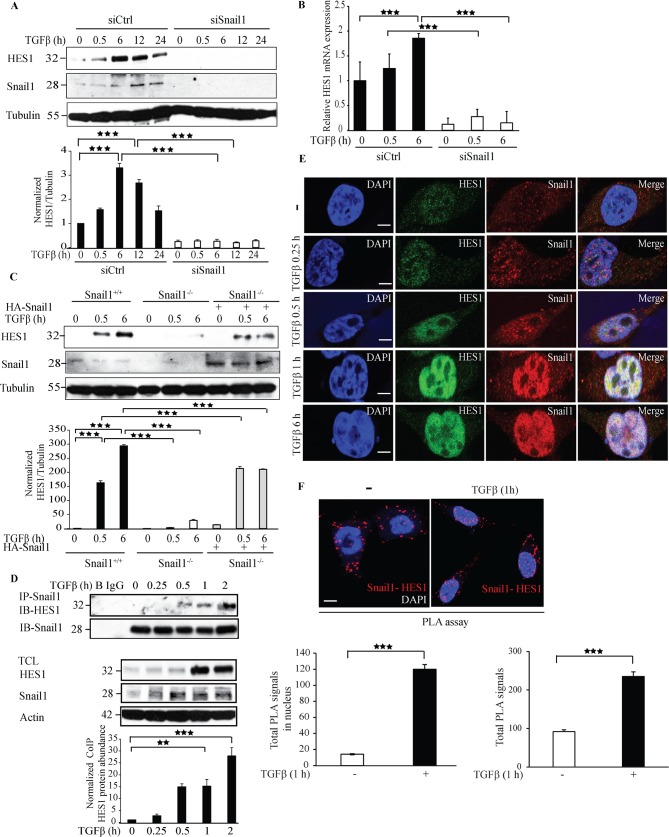
Knockdown of Snail1 expression by siRNA decreases Hes1 expression **A**. PC-3U cells were transiently transfected with control (Ctrl) or Snail1-specific siRNA #1 and treated with TGFβ (10 ng/ml) for the indicated time periods. Cell lysates were prepared and immunoblots were probed for Hes1, Snail1, and β-tubulin, which served as control for equal loading of proteins. (*n* = 5 independent experiments). **B**. qRT-PCR analysis of PC-3U cells transiently transfected with control (Ctrl) or Snail1-specific siRNA #1 and treated with TGFβ (10 ng/ml) as indicated. RNA was extracted and cDNA was prepared and used for qRT-PCR analysis of mRNA expression of Hes1. (*n* = 4 independent experiments). **C**. Snail1^+/+^, Snail1^−/−^ MEFs, and Snail1^−/−^ MEFs transiently transfected with HA-Snail1 were serum starved and treated with TGFβ for the indicated time periods. Cell lysates were prepared and immunoblots were probed for Hes1, Snail1, and β-tubulin, which served as control for equal loading of proteins (*n* = 4 independent experiments). **D**. PC-3U cells were treated with TGFβ for the indicated time periods. Cell lysates were prepared and immunoprecipitated with goat-Snail1 and immunoblotted with rabbit Hes1 antibodies. Total cell lysates were probed for endogenous Hes1, Snail1, and β-actin. (*n* = 4 independent experiments). **E**. Representative confocal imaging of PC-3U cells treated with TGFβ for the indicated time periods. Cells were fixed, permeabilised and incubated with anti-rabbit Hes1 and anti-mouse Snail1 antibodies, followed by incubation with Alexa Fluor 555 (red) secondary anti- mouse antibodies and Alexa Fluor 488 (green) secondary anti-rabbit antibodies for visualization. Merge of two layers shows co-localization of the proteins. Cell nuclei stained with DAPI. (*n* = 3 independent experiments). Scale bar, 20 μm. **F**. PLA images of PC-3U cells were treated with TGFβ for the indicated time periods. Cells were harvested, fixed, permeabilised and incubated with anti-rabbit Hes1 and anti-mouse Snail1 antibodies, followed by incubation with PLA probes. Hes1-Snail1 PLA complexes are visualized as red dots. Quantification of TβRI-Snail1 complexes was done with the aid of Blob finder software. (*n* = 5 independent experiments). Bar graphs show the means ± SEM; ^**^P < 0.005, ^***^P < 0.0005. Differences in the means ± SEM between samples were analyzed with two-way ANOVA and Bonferroni correction for multiple comparisons.

Hairy and enhancer of split-1 (Hes1) is an important transcriptional co-repressor implicated in Notch signaling. Interestingly, Hes1 has also been reported to play crucial role in EMT [38]. To investigate if Snail1 regulates Hes1 expression, we next silenced endogenous Snail1 expression by siRNA#1 or siRNA#2 and probed with antibodies specific to Hes1. Silencing Snail1 expression decreased Hes1 expression, suggesting that Snail1 enhances Hes1 expression (Figure 3A and Supplementary Figure 1A). qRT-PCR experiments confirmed that *Hes1* mRNA expression decreased in siSnail1 treated cells compared to siCtrl treated cells (Figure 3B). Moreover, we found that overexpression of HA-Snail1, or TGFβ stimulation of PC-3U cells, enhanced the expression of the transcriptional repressor Hes1 (Supplementary Figure 6A). Also, re-introduction of HA-Snail1 in Snail1^−/−^ MEFs rescued the expression of Hes1 (Figure 3C). Co-immunoprecipitation experiments further showed that TGFβ treatment for 1 h induced an interaction between endogenous Snail1 and Hes1 (Figure 3D). Confocal imaging of PC-3U cells treated with TGFβ for 1 h, revealed a co-localization of endogenous Hes1 and Snail1 (Figure 3E) Moreover, a PLA showed that TGFβ stimulation of cells significantly increased Hes1-Snail1 complexes in the nucleus (Figure 3F). These results suggest that Snail1 regulates Hes1 expression and that Snail1 interacts with Hes1 in the nucleus after 1 h of TGFβ treatment.

### Overexpression of HA-Snail1 promotes TβRI expression

To further evaluate our finding that Snail1 regulates TβRI expression, we overexpressed HA-Snail1 at increasing concentrations and investigated the amount of TβRI protein by immunoblotting with antibodies against TβRI. HA-Snail1 overexpression at increasing concentrations enhanced expression of TβRI-FL and also TβRI-ICD expression upon TGFβ stimulation (Supplementary Figure 6B). qRT-PCR data revealed that overexpression of HA-Snail1 or TGFβ stimulation of PC-3U cells, enhanced mRNA expression of TβRI (Supplementary Figure 6C).

The qRT-PCR data also supported the finding that TGFβ stimulation enhanced the mRNA expression of Hes1 at increasing concentrations of HA-Snail1 overexpression; however, at 6 μg concentration TGFβ stimulation decreased expression of TβRI and did not affect Hes1 expression (Supplementary Figure 6C, D). As expected, TGFβ stimulation enhanced the Snail1 mRNA expression (Supplementary Figure 6E).

### TβRI induces Hes1 expression

**Figure 4:**
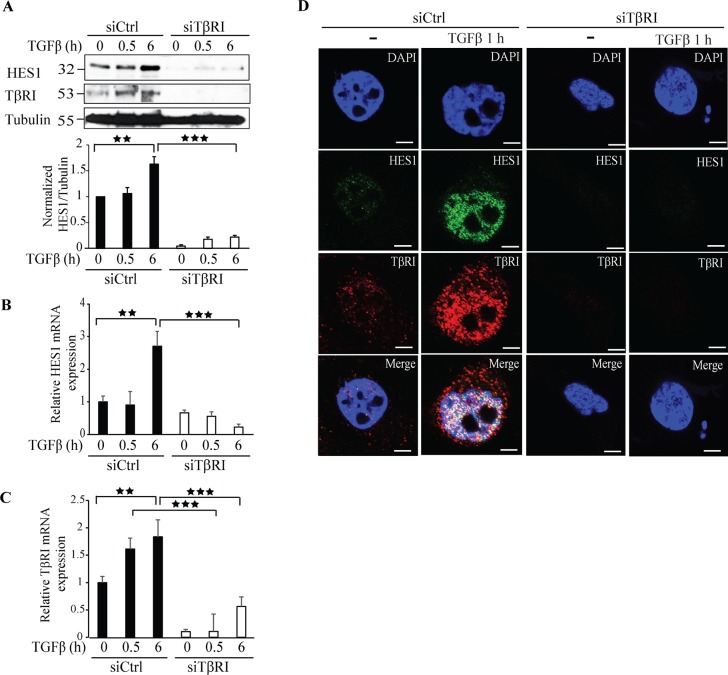
Knockdown of TβRI expression by siRNA decreases Hes1 expression **A**. PC-3U cells were transiently transfected with control (Ctrl) or TβRI-specific siRNA and treated with TGFβ (10 ng/ml) for the indicated time periods. Cell lysates were prepared and immunoblots were probed for Hes1, TβRI, and β-tubulin, which served as control for equal loading of proteins. (*n* = 5 independent experiments). **B**.-**C**. qRT-PCR analysis of PC-3U cells transiently transfected with control (Ctrl) or TβRI-specific siRNA and treated with TGFβ (10 ng/ml) as indicated. RNA was extracted and cDNA was prepared and used for qRT-PCR analysis of mRNA expression of Hes1 and TβRI. (*n* = 4 independent experiments). **D**. Confocal images of PC-3U cells transfected with control (Ctrl) or TβRI-specific siRNA. 48h post transfection, cells were incubated in low serum containing medium for 24 h followed by treatment with TGFβ (10 ng/ml) for the indicated time periods. Cells were fixed, permeabilised and incubated with anti-rabbit TβRI and anti-mouse Hes1 antibodies, followed by incubation with Alexa Fluor 555 (red) secondary anti-rabbit antibodies and Alexa Fluor 488 (green) secondary anti-mouse antibodies for visualization. Merge of two layers shows co-localization of the proteins. Cell nuclei stained with DAPI. (*n* = 3 independent experiments). Scale bar, 20 μm; Bar graphs show the means ± SEM; ^**^P < 0.005, ^***^P < 0.0005. Differences in the means ± SEM between samples were analyzed with two-way ANOVA and Bonferroni correction for multiple comparisons.

We reported previously that TβRI-ICD translocates to the nucleus, where it binds to the *Snail1* promoter [20]. To further explore other co-transcriptional targets regulated by TβRI-ICD, we silenced TβRI expression by siRNA and investigated the effect on the Notch responsive gene *Hes1*. Silencing TβRI expression decreased the expression of *Hes1* giving further evidence that *Hes1* is a TβRI responsive gene (Figure 4A). qRT-PCR experiments confirmed that downregulation of TβRI in PC-3U cells significantly decreased *Hes1* mRNA expression (Figure 4B, 4C). Moreover, treatment of PC-3U cells with siRNA specific for endogenous TβRI, decreased *Hes1* expression as visualized by confocal imaging (Figure 4D).

**Figure 5:**
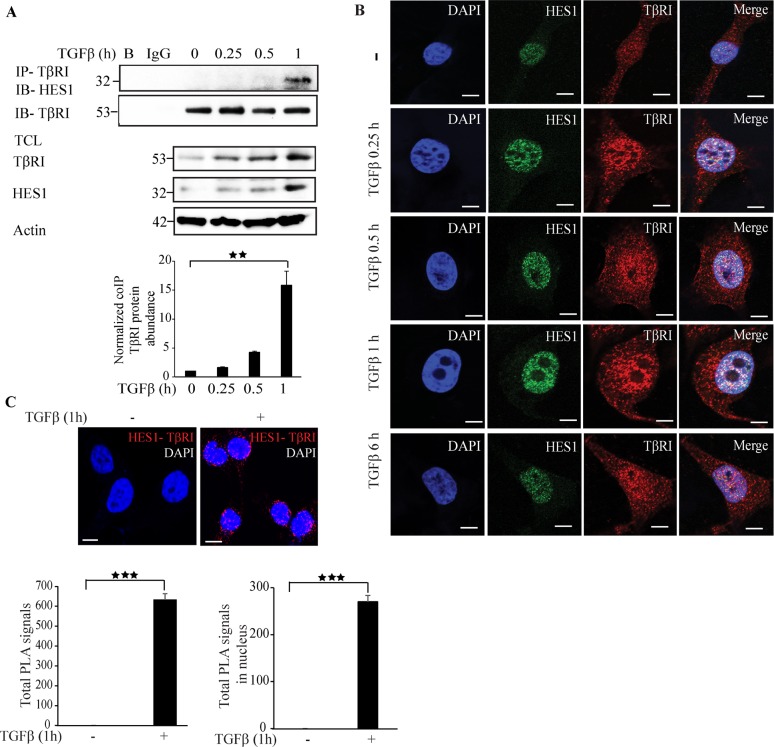
Hes1 interacts with TβRI **A**. PC-3U cells were treated with TGFβ for the indicated time periods. Cell lysates were prepared and immunoprecipitated with rabbit-TβRI and immunoblotted with rabbit-Hes1 antibodies. Total cell lysates were probed for endogenous TβRI, Hes1, and β-actin. (*n* = 4 independent experiments). **B**. Representative confocal imaging of PC-3U cells treated with TGFβ for the indicated time periods. Cells were fixed, permeabilised and incubated with anti-rabbit TβRI and anti-mouse Hes1 antibodies, followed by incubation with Alexa Fluor 555 (red) secondary anti-rabbit antibodies and Alexa Fluor 488 (green) secondary anti-mouse antibodies for visualization. Merge of two layers shows co-localization of the proteins. Cell nuclei stained with DAPI. (*n* = 3 independent experiments). Scale bar, 20 μm. **C**. PLA images of PC-3U cells were treated with TGFβ for the indicated time periods. Cells were fixed, permeabilised and incubated with anti-rabbit TβRI and anti-mouse Hes1 antibodies, followed by incubation with PLA probes. TβRI-Hes1 PLA complexes are visualized as red dots. Quantification of TβRI-Snail1 complexes was done with the aid of Blob finder software. (*n* = 3 independent experiments). Scale bar, 20 μm. Bar graphs show the means ± SEM; ^**^P < 0.005, ^***^P < 0.0005. Differences in the means ± SEM between samples were analyzed with two-way ANOVA and Bonferroni correction for multiple comparisons.

Co-immunoprecipitation experiments revealed that Hes1 and TβRI form a complex in a TGFβ dependent manner after 1 h of TGFβ stimulation of cells (Figure 5A). Confocal imaging supported this finding as TGFβ promoted formation of a complex between endogenous Hes1 and TβRI in the nucleus (Figure 5B). PLA assays also confirmed that Hes1 and TβRI interact, and that their interaction in the nucleus is enhanced upon TGFβ stimulation (Figure 5C). These results suggest that *Hes1* is a TβRI responsive gene and TGFβ promotes interaction and co-localization of TβRI and Hes1 in the nucleus.

### Treatment with TβRI kinase inhibitors decreases Hes1 expression

**Figure 6:**
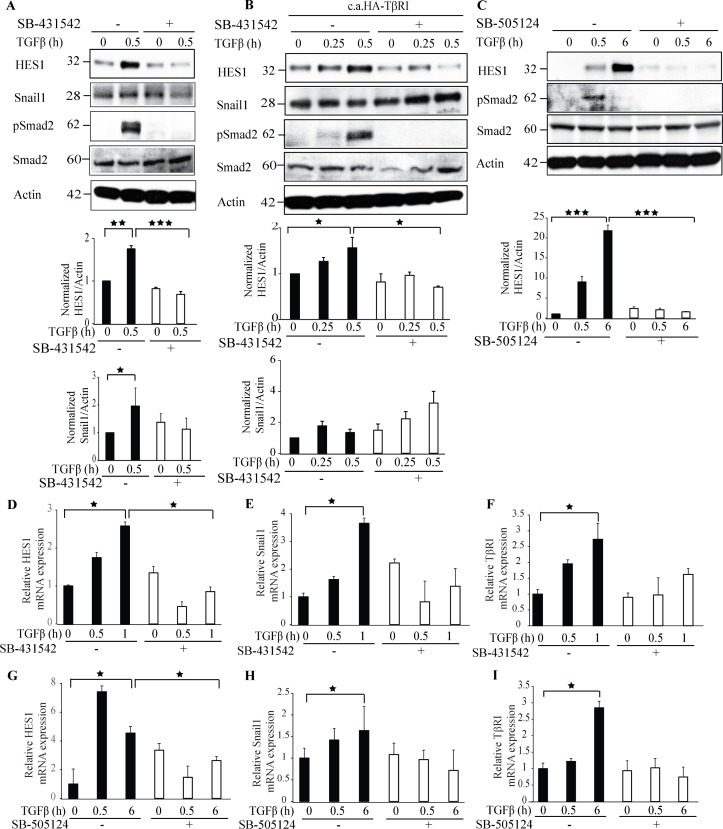
Treatment with TβRI kinase inhibitors decreases Hes1 expression **A**. PC-3U cells were treated with TGFβ for the indicated time periods. The cells were pretreated for 1h with TβRI kinase inhibitor (SB431542) before TGFβ treatment. Cell lysates were prepared and immunoblotted with Hes1, Snail1, p-Smad2, Smad2 and β-actin antibodies. (*n* = 4 independent experiments). **B**. PC-3U cells were transiently transfected with HA-TβRI and treated with TGFβ for the indicated time periods. The cells were pretreated for 1h with TβRI kinase inhibitor (SB431542) before TGFβ treatment. Cell lysates were prepared and immunoblotted with Hes1, Snail1, p-Smad2, Smad2 and β-actin antibodies. (*n* = 4 independent experiments). **C**. PC-3U cells were treated with TGFβ for the indicated time periods. The cells were pretreated for 1h with TβRI kinase inhibitor (SB505124) before TGFβ treatment. Cell lysates were prepared and immunoblotted with Hes1, Snail1, p-Smad2, Smad2 and β-actin antibodies. (*n* = 4 independent experiments). **D**.-**I**. qRT-PCR analysis of PC-3U cells pretreated with TβRI kinase inhibitors (SB431542 or SB505124) for 1h and stimulated with TGFβ as indicated. RNA was extracted and cDNA was prepared and used for qRT-PCR analysis of mRNA expression of Hes1, Snail1, TβRI. (*n* = 4 independent experiments). Bar graphs show the means ± SEM; ^*^P < 0.05, ^**^P < 0.005, ^***^P < 0.0005. Differences in the means ± SEM between samples were analyzed with two-way ANOVA and Bonferroni correction for multiple comparisons.

As we have observed that Hes1 and TβRI interact and form a complex in the nucleus, we investigated if the kinase activity of TβRI is required for Hes1 protein expression. Treatment with a commonly used and potent TβRI inhibitor; SB-431542 decreased the expression of Hes1 and, as expected, the phosphorylation of Smad2 (Figure 6A). Next, we ectopically overexpressed c.a. TβRI in PC-3U cells and treated them with the TβRI kinase inhibitor SB-431542. Consistent with our previous finding, Hes1 expression decreased upon treatment with the TβRI kinase inhibitor, when compared with untreated cells (Figure 6B). However, Snail1 expression was not affected by SB-431542 treatment (Figure 6B), in line with our previous reports that TβRI in the non-canonical signaling pathway, can promote transcription of Snail [21]. To validate our findings, we used also another TβRI inhibitor; SB-505124 (Figure 6C), and obtained similar results regarding Hes1 expression, as shown in Figure 6A. Taken together, these results suggest that the kinase activity of TβRI is required for Hes1 expression.

Next, we performed qRT-PCR experiments with RNA extracted from these samples. *Hes1* mRNA expression decreased significantly upon treatment with the TβRI kinase inhibitors SB-431542 or SB-505124 (Figure 6D, 6G); in contrast, *Snail1* and *TβRI* expression was partially decreased upon TGFβ stimulation (Figure 6E, 6H), (Figure 6F, 6I). These results further corroborate the notion that TβRI induces *Hes1* expression and the kinase activity of the TβRI is required for the regulation of Hes1 expression.

### Overexpression of HA-TβRI enhances Hes1 expression

Since we observed that silencing of TβRI decreased Hes1 expression, we next investigated if overexpression of HA-TβRI could affect regulation of Hes1. Results from qRT-PCR analysis, showed an enhanced expression of Hes1 upon TGFβ stimulation at increasing concentrations of HA-TβRI (Supplementary Figure 7A), suggesting that *Hes1* is a target gene of TβRI. However, the expression of Snail1 and Twist1 was slightly enhanced upon TGFβ stimulation at increasing concentrations of HA-TβRI, but not as much as in TGFβ-stimulated cells alone (Supplementary Figure 7B-7C). As expected, TGFβ stimulation enhanced the expression of TβRI mRNA (Supplementary Figure 7D). These results further strengthen the notion that TβRI regulates Hes1 expression.

### TβRI induces EMT genes

Since we observe that Snail1 regulates TβRI expression and since Snail1 is a bonafide master regulator of EMT genes, we investigated if the Snail1 regulated EMT genes are affected upon downregulation of TβRI. Treatment with TGFβ in the control cells upregulated mesenchymal genes, such as *Fibronectin1*, *N-cadherin*, the Notch responsive gene *Jagged1*, and *Zeb1*, compared to siTβRI treated cells (Supplementary Figure 8A-8D). As expected, the TβRI mRNA expression was downregulated in the siTβRI treated cells (Supplementary 8E) and also the classical TGFβ target genes; *Smad7* and *PAI-1* (Supplementary Figure 8F-8G). The mRNA expression of Snail1 also decreased in the siTβRI treated group (Supplementary Figure 8H). These results suggest that the Snail1 regulation of TβRI, in turn, promotes transcription of classical EMT genes.

### TGFβ stimulates sumoylation of Snail1

**Figure 7:**
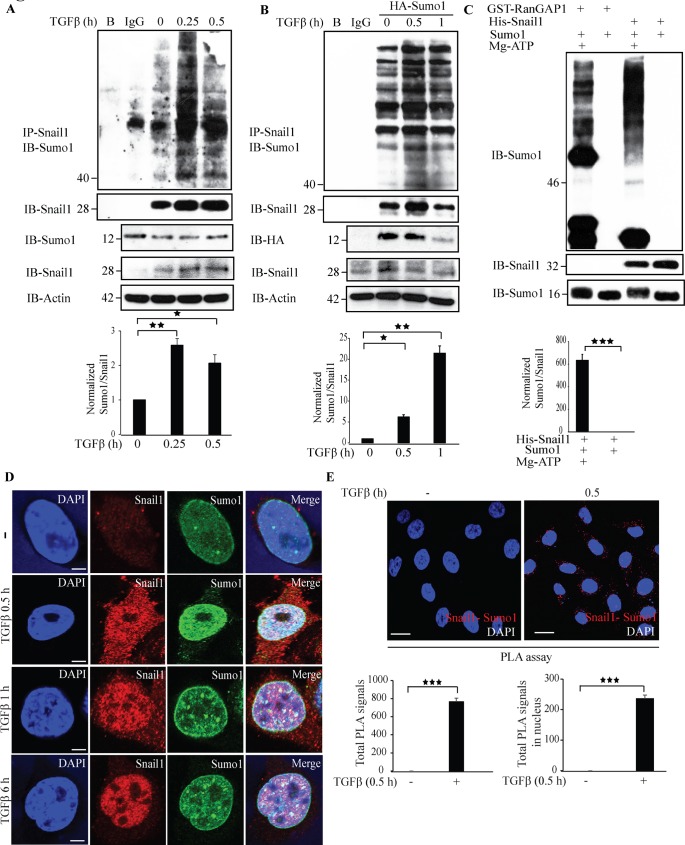
TGFβ stimulates sumoylation of Snail1 **A**. PC-3U cells were treated with TGFβ for the indicated time periods. Cell lysates were subjected to sumoylation assay and immunoprecipitated with goat-Snail1 and immunoblotted with rabbit-Sumo1 antibodies. Total cell lysates were probed for endogenous Sumo1, Snail1, and β-actin. (*n* = 5 independent experiments). **B**. PC-3U cells were transiently transfected with HA-Sumo1 and treated with TGFβ for the indicated time periods. Cell lysates were subjected to sumoylation assay and immunoprecipitated with goat-Snail1 and immunoblotted with rabbit-Sumo1 antibodies. Total cell lysates were probed for HA and endogenous Snail1, and β-actin. (*n* = 5 independent experiments). **C**. His-Snail1 and GST-RanGAP1 (positive control) incubated with Sumo1 was subjected to *in vitro* sumoylation reaction for 60 min in the presence or absence of Mg-ATP. Reaction was terminated by adding sample buffer and boiling at 95°C. Samples were immunoblotted with anti-Sumo1 and anti-Snail1 antibodies. (*n* = 6 independent experiments). **D**. Representative confocal imaging of PC-3U cells treated with TGFβ for the indicated time periods. Cells were fixed, permeabilised and incubated with anti-mouse Snail1 and anti-rabbit Sumo1 antibodies, followed by incubation with Alexa Fluor 555 (red) secondary anti-mouse antibodies and Alexa Fluor 488 (green) secondary anti-rabbit antibodies for visualization. Merge of two layers shows co-localization of the proteins. Cell nuclei stained with DAPI. (*n* = 3 independent experiments). Scale bar, 20 μm; **E**. PLA images of PC-3U cells were treated with TGFβ for the indicated time periods. Cells were harvested, fixed, permeabilised and incubated with anti-rabbit Sumo1 and anti-mouse Snail1 antibodies, followed by incubation with PLA probes. Sumo1-Snail1 PLA complexes are visualized as red dots. Quantification of Sumo1-Snail1 PLA complexes was done with the aid of Blob finder software. (*n* = 3 independent experiments). Scale bar, 20 μm. Bar graphs show the means ± SEM; ^*^P < 0.05, ^*^P < 0.005, ^***^P < 0.0005. Differences in the means ± SEM between samples were analyzed with two-way ANOVA and Bonferroni correction for multiple comparisons.

The activity and stability of proteins is regulated by post-translational modifications [39, 40]. To explore if Snail1 is sumoylated, we subjected lysates from untreated or TGFβ-treated PC-3U cells to immunoprecipitation with a Snail1 antiserum followed by immunoblotting with an antiserum against Sumo1. TGFβ treatment for 0.25 h induced a band at 40 kDa corresponding to polysumolyated Snail1 (Figure 7A). Next, we ectopically expressed HA-Sumo1 in PC-3U cells and treated with TGFβ, or not, and performed sumoylation assays. We observed a band at 40 kDa, which had the expected size of sumoylated Snail1 (Figure 7B). To further validate our findings, we performed *in vitro* sumoylation assays. Incubation of His-Snail1 with Sumo1 generated a band at 46 kDa, suggesting that His-Snail1 is sumoylated *in vitro* (Figure 7C). As a positive control RanGAP1 was used and found to be sumoylated in presence, but not in the absence of magnesium-ATP (Mg-ATP), which is essential for the conjugation of the substrate to the E1 activation enzyme and hence are derived from the SUMO cascade. These results showed that Snail1 was sumoylated by Sumo1 *in vitro* (Figure 7C). However, *in vitro* sumoylation assays with Sumo2 did not induce a product (Supplementary Figure 9). In addition, to investigate if sumoylation of Snail1 can be observed also in other cancer cells, we ectopically expressed HA-Sumo1 in breast cancer cells MDA-MB-231, and lung cancer cells A549. The cells were then treated with TGFβ, or not, and sumoylation assays were performed. TGFβ treatment of these cancer cells resulted also in sumoylation of Snail1, Supplementary Figure 10A, 10B. By using confocal imaging, we observed that TGFβ promotes a co-localization between Sumo1 and Snail1 in PC-3U cells (Figure 7D). Moreover, a PLA assay also showed that Snail1 and Sumo1 interact in a TGFβ-dependent manner in PC-3U, MDA-MB-231, and A549 cells (Figure 7E, Supplementary Figure 10C, 10D). Taken together, these results suggest that TGFβ stimulates sumoylation of Snail1, in aggressive prostate, breast and lung cancer cells.

### Lysine 234 is required for Sumo1 modification of Snail1

**Figure 8:**
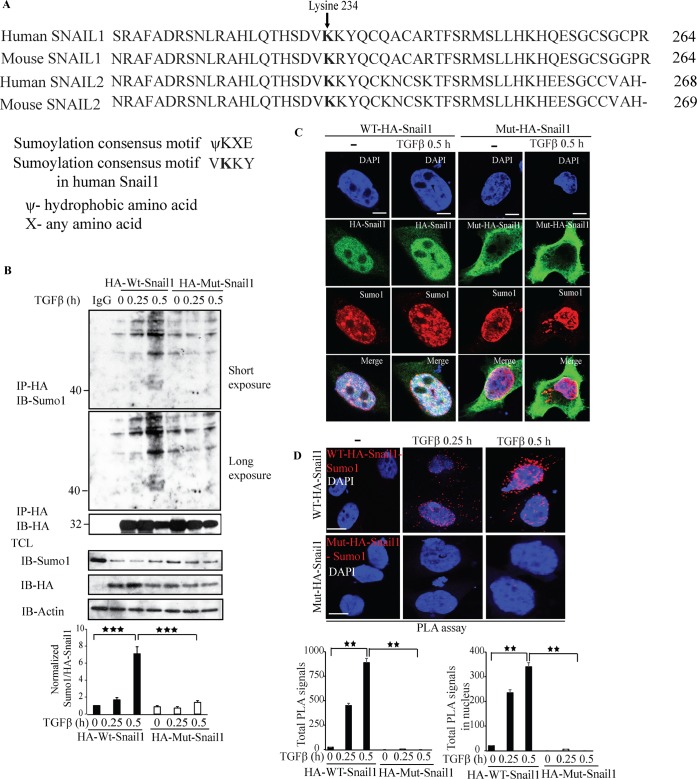
Lysine 234 is required for Sumo1 modification of Snail1 **A**. *In silico* analysis using Clustal W alignment of protein sequences of Snail1 and Snail2 (slug) of human and mice showing conserved lysine (K) residues. **B**. PC-3U cells were transiently transfected with HA-WT-Snail1 or Mutant-HA-Snail1 and treated with TGFβ for the indicated time periods. Cell lysates were subjected to sumoylation assay and immunoprecipitated with mouse-HA antibodies and immunoblotted with rabbit-Sumo1, mouse-HA antibodies. Total cell lysates were probed for endogenous Sumo1, HA, and β-actin. (*n* = 5 independent experiments). **C**. Representative confocal imaging of PC-3U cells transiently transfected with HA-WT-Snail1 or Mutant-HA-Snail1 and treated with TGFβ for the indicated time periods. Cells were harvested, fixed, permeabilised and incubated with anti-mouse HA-Snail1 and anti-rabbit Sumo1 antibodies, followed by incubation with Alexa Fluor 555 (red) secondary anti-rabbit antibodies and Alexa Fluor 488 (green) secondary anti-mouse antibodies for visualization. Merge of two layers shows co-localization of the proteins. Cell nuclei stained with DAPI. (*n* = 3 independent experiments). Scale bar, 20 μm. **D**. PLA images of PC-3U cells transiently transfected with HA-WT-Snail1 or Mutant-HA-Snail1 and treated with TGFβ for the indicated time periods. Cells were harvested, fixed, permeabilised and incubated with anti-rabbit Sumo1 and anti-mouse HA antibodies, followed by incubation with PLA probes. Sumo1-HA-Snail1 PLA complexes are visualized as red dots. Quantification of Sumo1- HA-Snail1 PLA complexes was done with the aid of Blob finder software. (*n* = 3 independent experiments). Scale bar, 20 μm. Bar graphs show the means ± SEM; ^*^P < 0.05, ^**^P < 0.005, ^***^P < 0.0005. Differences in the means ± SEM between samples were analyzed with two-way ANOVA and Bonferroni correction for multiple comparisons.

As we have observed that Snail1 is sumoylated upon TGFβ treatment, we investigated the possible acceptor lysine residue in Snail1 that is subjected to sumoylation. Using *in silico* analysis, we identified K234 as a possible acceptor lysine residue for Sumoylation of Snail1, (Figure 8A). Therefore, we generated a K234R mutant Snail1 by mutating K234 to arginine. We transiently transfected either wild type Snail1 (wt Snail1) or K234R mutant Snail1 in PC-3U cells and performed cell based sumoylation assays. Treatment with TGFβ induced a band corresponding to 40 kDa only in wt-Snail1 transfected group but not in cells transfected with K234R mutant Snail1 (Figure 8B), suggesting that K234R is a major acceptor lysine in Snail1 for sumoylation. By confocal imaging, we showed that Snail1 and Sumo1 co-localize and form a complex only in wt-Snail1 transfected cells but not in cells transfected with the K234R mutant Snail1 (Figure 8C), further confirming that the K234R is a crucial sumoylation site in Snail1. By PLA assays we observed that wt Snail1, but not the K234R mutant, interacted with Sumo1 in a TGFβ-dependent manner (Figure 8D). Moreover, qRT-PCR analysis revealed that wt-Snail1 promoted transcription of other EMT inducers, such as *Slug, Zeb1*, and *TβRI, c-Jun*, and TGFβ responsive genes such as *Smad7* and *PAI-1*, in transiently transfected PC-3U cells, but not K234R mutant (Supplementary Figure 11A-11F). These results suggest that lysine 234 is an acceptor residue for sumoylation of Snail1, as mutation of lysine 234 to arginine (K234R) abolished Snail1 sumoylation, and its capability to promote transcription of EMT genes.

### Sumoylation-deficient K234R mutant Snail1 does not promote migration and invasion

**Figure 9:**
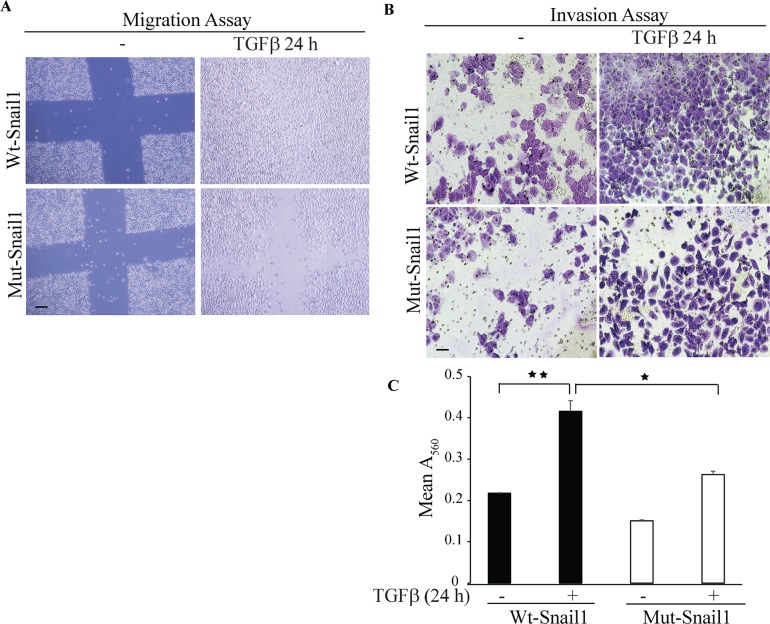
Sumoylation deficient Mut-Snail1 is deprived of migration and invasive properties **A**. *In vitro* wound healing assay was performed with PC-3U cells which were transiently transfected with HA-WT-Snail1 or Mutant-HA-Snail1. Thereafter, cells were starved in 1% FBS containing medium for 24 h and treated with TGFβ for the indicated time periods. Phase-contrast images were taken before and after treatment with TGFβ. Bar indicates 200μm. **B**. Representative images of invasive PC-3U cells transiently transfected with HA-WT-Snail1 or Mutant-HA-Snail1, treated with TGFβ as indicated. Invasive cells were visualized by staining with crystal violet cell stain solution. **C**. Graph represents mean values for optical density (OD) of invasive PC-3U cells transfected with HA-Snail1 or Mutant-HA-Snail1, and treated with or without TGFβ for the indicated time periods (*n* = 5 independent experiments). Bar graphs show the means ± SEM; ^*^P < 0.05, ^**^P < 0.005. Differences in the means ± SEM between samples were analyzed with two-way ANOVA and Bonferroni correction for multiple comparisons.

To investigate the functional properties of the sumoylation-deficient K234R mutant Snail1, we performed cell migration assays in PC-3U cells transfected with either wt-Snail1 or K234R mutant Snail1. Using scratch assays, in the presence or absence of TGFβ treatment, we found that wt Snail1, but not K234R mutant Snail1, promoted migration of PC-3U cells (Figure 9A).

As Snail1 is regarded as the master regulator of the EMT program and as invasiveness is considered to be a hallmark of cells undergoing EMT, we next performed invasive assays in PC-3U cells transfected with either wt or K234R mutant Snail1. TGFβ treatment for 24 h conferred invasiveness only in cells transfected with wt Snail1, but not in cells transfected with K234R mutant Snail1 (Figure 9B, 9C). These results further supports the notion that sumoylation deficient-Snail1 is unable to promote migration and invasion in prostate cancer cells.

### TGFβ promotes Snail1 and c-Jun interaction and co-localization

**Figure 10:**
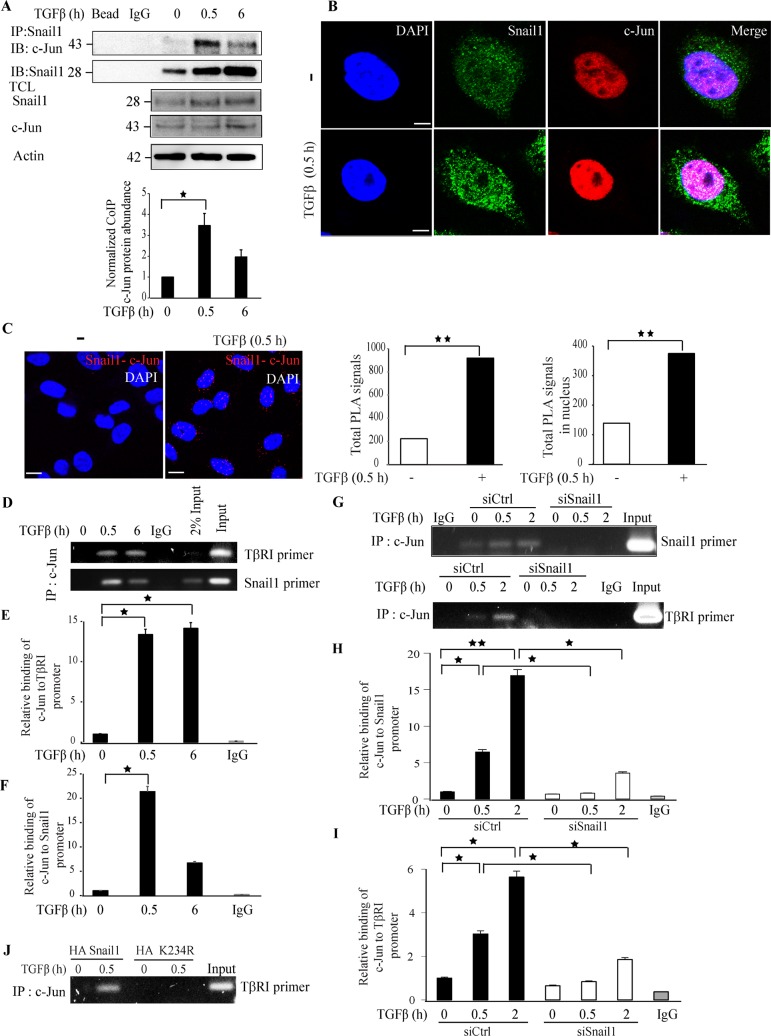
TGFβ promotes Snail1 and c-Jun interaction and co-localisation **A**. PC-3U cells were treated with TGFβ for the indicated time periods. Cell lysates were prepared and immunoprecipitated with goat-Snail1 and immunoblotted with rabbit-c-Jun antibodies. Total cell lysates were probed for endogenous Snail1, c-Jun, and β-actin. (*n* = 4 independent experiments). **B**. Representative confocal imaging of PC-3U cells treated with TGFβ for the indicated time periods. Cells were fixed, permeabilised and incubated with anti-rabbit c-Jun and anti-mouse Snail1 antibodies, followed by incubation with Alexa Fluor 555 (red) secondary anti-rabbit antibodies and Alexa Fluor 488 (green) secondary anti-mouse antibodies for visualization. Merge of two layers shows co-localization of the proteins. Cell nuclei stained with DAPI. (*n* = 3 independent experiments). **C**. PLA images of PC-3U cells were treated with TGFβ for the indicated time periods. Cells were fixed, permeabilised and incubated with anti-rabbit c-Jun and anti-mouse Snail1 antibodies, followed by incubation with PLA probes. c-Jun-Snail1 PLA complexes are visualized as red dots. Quantification of c-Jun-Snail1 PLA complexes was done with the aid of Blob finder software. (*n* = 3 independent experiments). **D**. Chromatin immunoprecipitation (ChIP) assay of c-Jun binding to the TβRI and Snail promoters. PC-3U cells treated or not with TGFβ as indicated and ChIP samples were subjected to agarose gel electrophoresis (*n* = 5 independent experiments). **E**. qRT-PCR analysis of relative binding of c-Jun to TβRI promoter. **F**. qRT-PCR analysis of relative binding of c-Jun to Snail1 promoter. **G**. PC-3U cells transiently transfected with control (Ctrl) or Snail1-specific siRNA #1 and treated with TGFβ (10 ng/ml) for the indicated time periods. ChIP samples were subjected to agarose gel electrophoresis (*n* = 5 independent experiments). **H**. qRT-PCR analysis of relative binding of c-Jun to Snail1 promoter. **I**. qRT-PCR analysis of relative binding of c-Jun to TβRI promoter. **J**. PC-3U cells transiently transfected with HA-Snail1 or Mutant-HA-K234R-Snail1 and treated with TGFβ (10 ng/ml) for the indicated time periods. ChIP samples were subjected to agarose gel electrophoresis to analyze relative binding of c-Jun to TβRI promoter (*n* = 5 independent experiments). Bar graphs show the means ± SEM; ^*^P < 0.05, ^**^P < 0.005, ^***^P < 0.0005. Differences in the means ± SEM between samples were analyzed with two-way ANOVA and Bonferroni correction for multiple comparisons.

We have previously reported that c-Jun binds to the Snail1 promoter and regulates Snail1 expression. We investigated if Snail1 and c-Jun interact, by performing an endogenous co-immunoprecipitation assay; treatment with TGFβ enhanced interaction between Snail1 and c-Jun (Figure 10A). Confocal imaging and PLA assays also supported the notion that Snail1 and c-Jun co-localize and interact in the nucleus in a TGFβ-dependent manner (Figure 10B). Interestingly, only wt-Snail1 interacted with c-Jun, while the sumoylation-deficient K234R mutant Snail1 did not, suggesting that sumoylation of Snail1 is required for interaction between Snail1 and c-Jun (Supplementary Figure 12). PLA assays also supported our finding that Snail1 and c-Jun interact in a TGFβ-dependent manner (Figure 10C).

Next, we confirmed our previous finding that c-Jun binds to Snail1 promoter by ChIP assays and explored the possibility that c-Jun binds to the TβRI promoter [30]. Treatment of PC-3U cells with TGFβ enhanced c-Jun binding to TβRI and Snail1 promoters (Figure 10D-10F), suggesting that c-Jun binds to both Snail1 and TβRI promoters to regulate their expression. Moreover, knockdown of endogenous Snail1 protein expression inhibited the binding of c-Jun to the Snail1 and TβRI promoters (Figure 10G-10I), suggesting that Snail1 protein facilitates binding of c-Jun to the Snail1 promoter. Taken together, Snail1 interaction with c-Jun promotes TGFβ-dependent binding of c-Jun to the Snail1 promoter. In addition, by Chip assays we found that c-Jun binds to the TβRI promoter only in the presence of wt-Snail1, but not in the presence of the K234R mutant Snail1, suggesting that K234 is crucial for active gene regulation (Figure 10J).

### Immunohistochemical stainings reveal a correlation between Snail1, Sumo1, TβRI and c-Jun expression and malignancy in prostate cancer tissues

**Figure 11:**
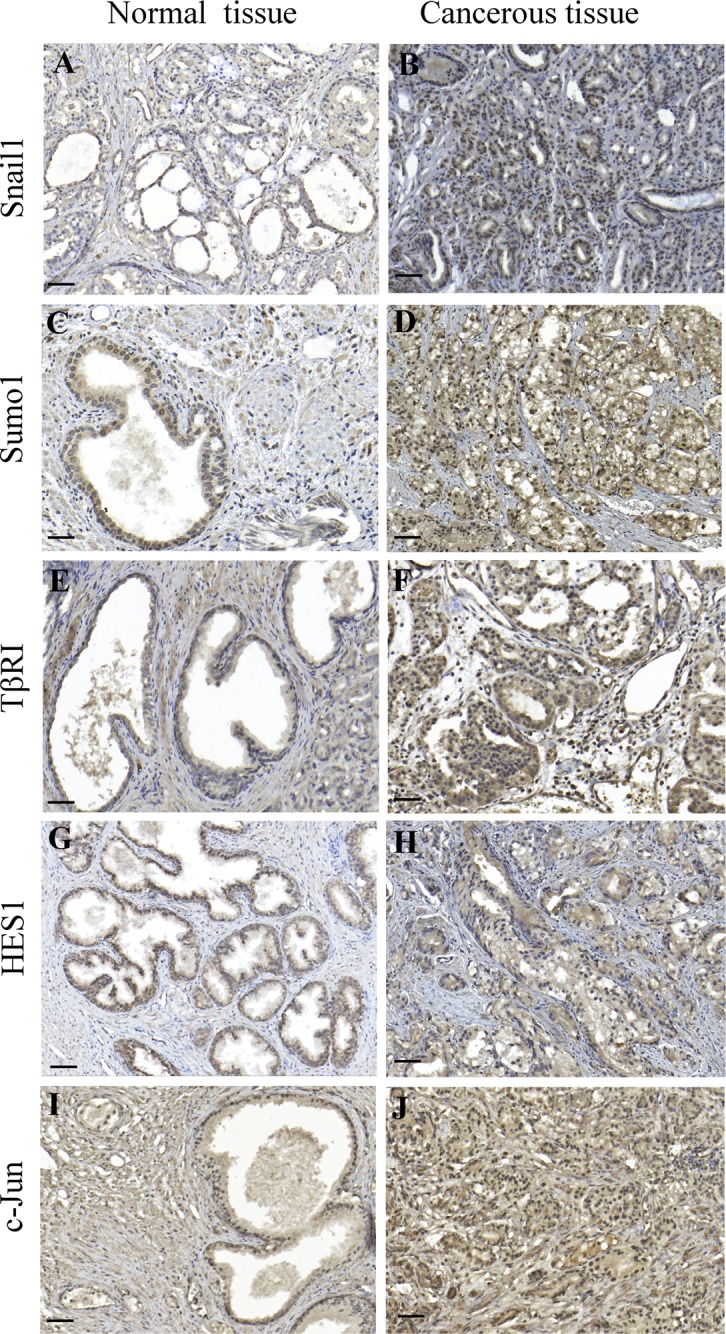
Immunohistochemical staining's for Snail1, Sumo1, TβRI, Hes1, and c-Jun correlates with malignancy of prostate cancer tissues **A**.-**J**. paraffin-embedded sections of prostate cancer tissues were immunostained with Snail1, Sumo1, TβRI, Hes1, and c-Jun antibodies. 3,3′-Diaminobenzidine was used as chromogen and Mayer hematoxylin as counterstain. Representative images from normal prostate glands (A,C,E,G,I) and cancer tissues (B,D,F,H,J: Scale bar 200 μm).

As we have observed that Snail1 is critical for EMT regulation in prostate cancer, we performed immunohistochemical stainings for Snail1, Sumo1, TβRI, Hes1 and c-Jun, in prostate cancer tissues. Increased positive stainings for Snail1, Sumo1, TβRI, Hes1, and c-Jun were found in the investigated prostate cancer tissues compared to the normal tissues (Figure 11A-11J), suggesting that TGFβ-promoted prostate cancer progression, involves increased expression of these EMT promoting factors. No immunohistochemical stainings for Snail1, Sumo1, TβRI, Hes1 and c-Jun was observed in control experiment when the primary antibodies were omitted) (Supplementary Figure 13A-13E).

## DISCUSSION

The bifunctional role of TGFβ as a tumor suppressor and tumor promoter is well established [2, 41]. TGFβ dictate cellular functional responses by activation of canonical Smad and non-Smad signaling cascades [1, 7, 16]. Previously, we reported that TβRI is cleaved in the ectodomain by TACE and in the transmembrane region by presenilin1 to liberate an TβRI-ICD [20, 21, 36, 42]. The liberated TβRI-ICD translocates to the nucleus where it binds to the *Snail1* promoter to promote invasion of cancer cells.

*Snail1* is a pro-invasive gene regulated by both canonical and non-canonical TGFβ signaling members [26, 30]. Although *Snail1* has been reported to be upregulated by TGFβ to promote EMT, the downstream targets for *Snail1* are not fully understood and was the topic for our current investigation. In our quest to unravel possible downstream regulators of Snail1, we silenced the expression of Snail1 by siRNA and found that TβRI expression decreased in the absence of Snail1, suggesting that Snail1 regulates TβRI expression (Figure 1, Supplementary Figure 1) and that treatment of prostate cancer cells with TGFβ stimulated formation of Snail1 and TβRI-ICD complexes in the nucleus of these cancer cells (Figure 2, Supplementary Figures 2 and 4). Snail1 has previously been reported to interact with the R-Smad, Smad3 and co-Smad, Smad4 to drive EMT in breast carcinomas [27]. In this context, Snail1 interaction with TβRI and regulation of TβRI expression, is a novel mechanism identified in this report by which Snail1 further enhance TGFβ signaling and EMT in prostate cancer.

Hes1 is a key transcriptional target in the notch signaling cascade and has also been implicated to play a crucial role in cancer metastasis, and EMT [38, 43, 44] and elevated Jagged 1 expression has been reported in prostate cancer metastasis [45–48]. As we have previously observed that the TβRI-ICD forms a complex with the Notch-ICD (NICD) in the nucleus [36], we investigated the possibility if other Notch targets such as *Hes1*, is regulated by TβRI-ICD. Interestingly, downregulation of Snail1 expression caused a decreased Hes1 expression, suggesting that Hes1 is also regulated by Snail1 (Figure 3). Since we observed that Snail1 downregulation decreases both TβRI and Hes1 expression, we also investigated if TβRI is the upstream regulator of Hes1. siTβRI treatment decreased the expression of Hes1, suggesting that Hes1 is a TβRI target gene (Figure 4) and also other EMT genes (Supplementary Figure 8). TGFβ treatment induced interaction between Hes1 and TβRI (Figure 5), suggesting that nuclear translocated TβRI-ICD interacts with Hes1. Our finding in this report shows that the TβRI-ICD interacts with Hes1 and that TβRI regulates Hes1 and Jagged1 expression. The current data, along with data from our previous study, in which we found that TβRI-ICD and NICD interact [36], enhances our knowledge about the common transcriptional targets of the TGFβ and the Notch signaling cascades [22, 36]. Moreover, the kinase activity of TβRI is crucial for regulating the expression of Hes1 but not for Snail1 expression (Figure 6). As we previously have reported that Snail1 expression is independent of TβRI kinase activity, Snail1 might be regulated by other signaling cascades such as NF-κB, Ras, PI3K/Akt and FGF, as it has been reported that Snail1 promoter consists of transcriptional binding sites for the aforementioned signaling pathway members [30, 31, 49].

Sumoylation of various proteins has been reported to regulate gene transcription, nuclear transport, and activation of signal transduction pathways [33]. In our study, we have elucidated that TGFβ stimulates Snail1 sumoylation (Figures 7, 8 and Supplementary Figure 10) at lysine 234. Mutation of Snail1 at lysine 234 to arginine (K234R) inhibited sumoylation of Snail1, its nuclear translocation, and also the TGFβ-induced migratory and invasive properties in prostate cancer cells (Figure 9), suggesting that sumoylation is important for Snail1 function.

Polyubiquitination of Snail1 by the ubiquitin ligase FBXL5 has been reported to disrupt its ability to bind to DNA [32]. Moreover, K234 in Snail1 has also been identified as one of the three acceptor lysine residues (K85, K146, K234) for ubiquitination. However K234 is modified upto a very less extent compared to full length Snail1 protein by FBXL5 and the authors also suggested that this ubiquitination modification may not lead to degradation, compared to K146, which is known to be involved in Snail1 proteosomal degradation both by β-TrCP1 and FBXL14, by K48-polyubiquitnation [32, 50]. Snail1 has a conserved nuclear localization signal (NLS) and is recognized by the importins (β, α and 7) and transportin for active transport from cytoplasm to the nucleus [51, 52]. Moreover, lysine 234 has been recognized as one among several residues important in Snail1 for DNA binding [51]. In this context, sumoylation of Snail1 may be a crucial event for its nuclear translocation, as it could be speculated that this modification may lead to conformational changes in the structure of Sumo1, thereby giving access for importins to bind and promote its nuclear transport.

Snail1 has been reported to bind to its own promoter and to regulate its own expression [29]. Previously, we reported that TGFβ induced transcription of Snail1 requires *c-Jun* and *c-Jun* binds to specific distal region of the Snail1 promoter [30, 31]. Immunoprecipitation experiments confirmed that TGFβ stimulates interaction and co-localization between Snail1 and *c-Jun* (Figure 10). We report here that *c-Jun* binds to TβRI and Snail1 promoter to regulate their expression respectively. Moreover, TGFβ induced Snail1 protein is crucial for *c-Jun* to bind to TβRI and Snail1 promoters (Figure 10), suggesting that the Snail1 transcription factor might recruit other co-activators such as c-Jun, to bind to TβRI or to its own promoter region. Interestingly, only the wt-Snail1 promoted *c-Jun* binding to TβRI promoter, compared to the Lysine 234 mutant Snail1, to promote transcription of EMT genes, including *TβRI* and *c-Jun* (Figure 10, Supplementary Figure 11, 12).

Given that Snail1 is a key regulator of the EMT program, we also investigated the mRNA expression profile of other EMT regulator genes, such as *Zeb1*, *Slug*, and *Twist1*. Knockdown of endogenous Snail1 expression decreased expression of all three regulator genes, suggesting that Snail1 is a key inducer of EMT (Supplementary Figure 5D-5F). Moreover, Snail1 enhanced the expression of mesenchymal markers, such as *Fibronectin1, N-cadherin*, and *Jagged1* (Supplementary Figure 5A-5C). Taken together, these results suggest that Snail1 is the master regulator of EMT, controlling the expression of EMT genes and interestingly, also TβRI, as demonstrated in this report. Remarkably, knockdown of endogenous TβRI expression decreased expression of *Fibronectin1, N-cadherin*, and the Notch responsive target *Jagged1*, the EMT regulator *Zeb1* (Supplementary Figure 8A-8D), the classical TGFβ targets *Smad7* and *PAI-1* (Supplementary Figure 8F-8G). Taken together, our data suggest that Snail1-regulated TβRI promotes the mesenchymal phenotype and enables the cancer cells to become invasive.

Androgen receptors, which are known to be crucial for prostate cancer tumorigenesis, has been reported to be sumoylated [53–55]. In this context, our data propose that the androgen-independent PC-3U cells, instead utilize sumoylated Snail1 to target TβRI to promote prostate cancer progression, as we have observed overexpression of Snail1, Sumo1, TβRI, Hes1, and c-Jun in prostate cancer specimens (Figure 11). These results suggest that Snail1 and Sumo1 might be markers for prostate cancer progression. As sumoylation inhibitors are currently being tested to combat breast cancer tumorigenesis [55], it would be interesting to target Snail1 sumoylation to inhibit migration and invasion in prostate cancer, as we in this report identify sumoylated Snail1 as a crucial modulator of the EMT program in androgen independent aggressive prostate cancer cells and also in the highly invasive breast cancer (MDA-MB-231) and lung cancer cells (A549).

## MATERIALS AND METHODS

### Cell culturing

Human prostate PC-3U cancer cells, a sub-cell line of PC3, have been previously described [36]. These cells were grown in RPMI-1640 medium supplemented with 10% fetal bovine serum (FBS), 1% L-glutamine, and 1% penicillin and streptomycin. Cells were starved in RPMI-1640 medium supplemented with 1% FBS, 1% L-glutamine, and 1% penicillin and streptomycin. Snail1^+/+^ or Snail1^−/−^ mouse embryo fibroblasts (MEFs) were a kind gift from Dr. Antonio García de Herreros, Barcelona), MDA-MB-231, A549 cells were cultured in DMEM medium containing 10% fetal bovine serum (FBS), 1% L-glutamine, and 1% penicillin and streptomycin.

Cells were starved in DMEM medium containing 0.5% FBS, 1% L-glutamine, and 1% penicillin and streptomycin. After starvation, cells were stimulated with 10 ng/ml of TGFβ1 ligand (R&D Systems), and then harvested by washing with phosphate-buffered saline (PBS) and lysed in RIPA lysis buffer containing (50 mM Tris (pH 8.0), 150 mM NaCl, 1% Triton X-100, 10% (v/v) glycerol, 1 mM aprotinin, 1 mM Pefabloc, 1 mM sodium orthovanadate). Protein concentration was measured using the BCA protein assay kit (Nordic Biolabs), and equal amounts of protein were loaded onto sodium dodecyl sulfate-polyacrylamide gel electrophoresis (SDS-PAGE) using gels from Invitrogen. After electrophoresis, proteins were transferred onto a nitrocellulose membrane using an I-blot machine (Invitrogen), blocked in 5% bovine serum albumin (BSA) and incubated with the respective antibodies.

### Antibodies

Rabbit antisera against HA, p-Smad2, Smad2, Sumo1, Hes1, β-tubulin, and a mouse monoclonal against HA, were purchased from Cell Signaling; rabbit antisera against TβRI (V22) and Snail1-were from Santa Cruz Biotechnology and Novus Biologicals, respectively; a goat antiserum against Snail1, from RnD systems; a mouse antiserum against Hes1 was from Abcam; a mouse antiserum against β-actin from Sigma Aldrich; and secondary horseradish peroxidase–conjugated goat anti-rabbit, goat anti-mouse, and anti-goat antibodies were from DAKO. Light-chain specific anti-rabbit, anti-mouse, and anti-goat antibodies were purchased from Jackson Laboratories.

### Plasmids

HA-tagged constitutively active (c.a)TβRI (HA-TβRI) and Flag-tagged c.aTβRI was a kind gift from Prof. P. ten Dijke, Leiden. Green fluorescent protein (GFP)-tagged c.aTβRI (GFP-TβRI) was constructed in-house and has been previously described [20]. HA-Snail1 was kindly provided by Prof. A. Moustakas, Uppsala. GST-Snail1 and HA-Snail1 was provided by Dr. Antonio García de Herreros, Barcelona. HA-Sumo1 was a kind gift from Prof. I. Dikic, Frankfurt.

### Transfections

Transient transfections of ectopically expressed HA-TβRI or HA-Snail1 were performed with FuGENE® 6 transfection reagent (Promega) following the manufacturer's instructions. Transient transfections of small-interfering (si) RNA were performed using Oligofectamine reagent (Invitrogen) following the manufacturer's instructions. siRNA targeting TβRI, Snail1, or non-targeting control siRNA were purchased from Dharmacon, and Stealth siRNA specific to Snail1 and non-targeting control siRNA were obtained from Invitrogen.

### Site-directed mutagenesis

QuickChange Site-Directed Mutagenesis kit (Agilent Technologies) was used to create a K234R mutant HA-Snail1 plasmid, following the manufacturer's instructions. Mutagenesis was confirmed by sequencing of the plasmid.

### Luciferase reporter assay

Transient transfections of small-interfering (si) RNA were performed using Oligofectamine reagent (Invitrogen), following the manufacturer's instructions. Fortyeight hours after siRNA transfection, cells were transiently transfected with CAGA_12_ reporter and renilla constructs (internal control) with FuGENE® 6 transfection reagent (Promega) following the manufacturer's instructions. After starvation, cells were stimulated with TGFβ for the indicated time periods. Cells were harvested and lysed in lysis buffer (Promega). Triplicates of each sample were used for measuring the luciferase activity using a luminometer. Values were calculated as follows:

Luciferase activity of sample = CAGA_12_ reporter activity / renilla reporter activity.

### Immunoprecipitation assays and western blotting

After cells were stimulated for the indicated time periods, they were washed with PBS and lysed in cold RIPA buffer for 20 min at 4°C and centrifuged at 13,000 rpm for 10 min. The supernatant was taken, and the protein concentration was measured with the BCA protein assay kit (Nordic Biolabs) following the manufacturer's instructions. For immunoprecipitation, samples were incubated with primary antibody overnight; the next day, samples were incubated with Protein G Sepharose beads (GE Healthcare) at 4°C. Samples were washed with RIPA buffer three times and the proteins were extracted from the beads in SDS sample buffer containing reducing agent and heating at 95°C. Immunoblotting was performed by subjecting equal amount of protein from each treatment group to SDS-PAGE using 10% or 12% Bis-tris polyacrylamide gels (Invitrogen). Gels were run in MOPS buffer (Invitrogen), and protein was transferred onto a nitrocellulose membrane using an iBlot Machine (Invitrogen). Quantification of immunoblots were performed with Biorad Quantity one software. Values represent the means of three or more independent biological experiments. Bars are presented as means ± SEM and statistical analysis was based on at least three independent biological experiments. Differences in the means ± SEM between samples were analyzed with two-way ANOVA with bonferroni post hoc test using SPSS software.

### Immunofluorescence and confocal microscopy

PC-3U cells were cultured in six-well dishes in the presence of RPMI-1640 medium supplemented with 10% FBS, 1% L-glutamine, and 1% penicillin and streptomycin. Cells were transfected with the respective plasmids; after 24 h, cells were starved in RPMI-1640 medium supplemented with 1% FBS, 1% L-glutamine, and 1% penicillin and streptomycin, and after an additional 18 h, cells were stimulated with 10 ng/ml of TGFβ1.

Cells were washed in PBS and fixed in 4% paraformaldehyde, followed by permeabilization in 2% Triton X-100. Cells were blocked in 5% BSA. After blocking, cells were incubated with primary antibody for 1 h, followed by incubation with secondary donkey anti-rabbit Alexa Fluor 555 antibodies or goat anti-mouse Alexa Fluor 488 antibodies (Invitrogen). After staining, cells were mounted in medium with 4’,6-diamidino-2-phenylindole (DAPI) to visualize cell nuclei. Images were taken with a confocal microscope with an oil immersion 63X lens. Zen software was used to analyze the data.

### Proximity Ligation Assay (PLA)

PC-3U cells, untreated or treated with TGFβ, were fixed, permeabilized, blocked and probed with the primary antibodies as indicated, following the manufacturers protocol. Antibodies from different species against TβRI (V22), Snail1, Hes1, Sumo1 and c-Jun, were used. Next, the cells were incubated with PLA probes (Sigma-Aldrich), followed by ligation and amplification. Slides were mounted with Duolink mounting medium and evaluated with a confocal microscope (Carl Zeiss). Images were acquired with a 63X objective. Representative results are shown from experiments repeated at least three times. Cell images were exported in TIF format with Zen software (Carl Zeiss) for further analysis; quantification of PLA signals were determined with Blob-Finder image analysis software, which was developed by the Center for Image Analysis, Uppsala University, Sweden.

### RNA isolation, cDNA synthesis, and RT-PCR

RNA isolation was performed using the RNeasy Mini Kit (Qiagen), following the manufacturer's instructions. A total of 2 μg of RNA was used for cDNA synthesis using the Thermoscript cDNA synthesis kit (Invitrogen). qRT-PCR was performed using the Power SYBR Green Master Mix (Applied Biosystems). Samples were run on an Applied Biosystems 7900HT Fast Real-Time PCR system (Table 1).

**Table 1:** Primers used for qRT-PCR

Gene name	Forward primer	Reverse primer
*Snail1*	GAAAGGCCTTCAACTGCAAA	TGACATCTGAGTGGGTCTGG
*TβRI*	TGTTGGTACCCAAGGAAAGC	CACTCTGTGGTTTGGAGCAA
*Smad7*	TCCTGCTGTGCAAAGTGTTC	TCTGGACAGTCTGCAGTTGG
*PAI-1*	CTCTCTCTGCCCTCACCAAC	GTGGAGAGGCTCTTGGTCTG
*Hes1*	TGAAGAAAGATAGCTCGCGG	GGTACTTCCCCAGCACACTT
*Twist1*	TTCTCGGTCTGGAGGATGGA	CCCACGCCCTGTTTCTTTGAA
*Fibronectin1*	CCGTGGGCAACTCTGTC	TGCGGCAGTTGTCACAG
*N-Cadherin*	CGGCCCGCTATTTGTCATCA	TGCGATTTCACCAGAAGCCT
*Jagged1*	GATGATGGGAACCCGATCAA	GCAAGGGAACAAGGAAATCTGT
*Zeb1*	GCAGGTGAGCAACTGGGAAA	ACAAGACACCGCCGTCATTT
*Slug*	TGTTGCAGTGAGGGCAAGAA	GACCCTGGTTGCTTCAAGGA
*GAPDH*	TGATGACATCAAGAAGGTGGTGAAG	TCCTTGGAGGCCATGTGGGCCAT

### Chip assay

Chromatin immunoprecipitation (ChIP) assay was performed using the SimpleChIP® Plus Enzymatic Chromatin IP Kit from Cell Signaling Technology following the manufacturer's protocol. DNA and proteins were cross-linked in 4% formaldehyde. After shearing of the cells the chromatin was precipitated using c-Jun ChIP grade antibody, and reverse crosslinked. After purification, DNA was amplified using standard PCR and run on 1.5% agarose gel or subjected to quantitative RT-PCR with the Power SYBR PCR Master Mix.

The following ChIP primers were used (Table 2).

**Table 2:** ChIP primers

Gene name	Forward primer	Reverse primer
c-Jun	AAGCACACTTCCCTTTGCAT	GGACAGAACACTCAGAGCCT
TβRI	TGGAGCGTCTCGCAGTAAAT	CCCTTCTTAGCACCCAGCTC

### *In vitro* sumoylation assay

His-Snail1 (RnD Systems) and GST-RanGAP1 (Enzo Life Sciences) were used as substrates in i*n vitro* sumoylation assays, assays were performed following the instructions provided by the manufacturer (Enzo Life Sciences). His-Snail or GST-RanGAP1 (positive control) were incubated with Sumo E1 (Aos1 and Uba2), E2 (Ubc9), E3 Sumo1 or E3 Sumo2 enzymes in the presence or absence of Mg-ATP in sumoylation buffer for 1 h at 37°C. The reactions were quenched by adding SDS sample buffer and heating at 95°C for 5 min. The reaction mixture was analyzed by immunoblotting using anti-Sumo1, anti-Sumo2 and anti-Snail1 antibodies.

### *In vivo* sumoylation assay

PC-3U, MDA-MB-231, A549 cells were transfected with HA-Sumo1 or non-transfected cells, were stimulated with TGFβ for the indicated time periods [37], then harvested and washed in PBS. Cells were pelleted by centrifugation and incubated with 1% SDS, followed by heating at 95°C for 10 min. PBS containing 0.5% NP40 and proteinase inhibitors was added, followed by centrifugation at 13,000 rpm for 10 min. The supernatant was transferred to a new tube discarding the slimy layer, and immunoprecipitated with Snail1 antibodies, and analyzed by immunoblotting using anti-Sumo1 and anti-HA antibodies.

### Wound healing assays

Scratch wound healing assays using PC-3U cells were performed in 6-well plates. Cells were transfected with either wt-Snail1 or mutant-Snail1. Cells were serum-starved prior to creating an approximately 0.6 mm wide wound using a pipette tip. Thereafter, cells were stimulated with TGFβ for 24 h. Images were taken by a microscope with a digital camera, using QED software.

### Invasion assay

Invasion assays were performed using Corning BioCoat Growth Factor (GFR) reduced Matrigel Invasion Chamber, following the manufacturer's instructions. Serum-free RPMI-1640 was used to rehydrate the GFR Matrigel inserts. Cells were seeded into the insert in RPMI-1640 medium with 1% FBS, with or without TGFβ1. The lower chamber was filled with RPMI 1640 with 10% FBS. After 24 h of incubation in a humidified tissue culture incubator, non-invasive cells were scrubbed off the upper surface of the membrane. The remaining invasive cells were stained with crystal violet staining solution and then photographed. Colorimetric quantification was performed by measuring the optical density at 560 nm using a spectrophotometer.

### Immunohistochemistry

Paraffin embedded tissue sections from patients with prostate cancer, were deparaffinised and tissue sections were prepared according to the manufacturer's instructions (Thermoscientific). Primary antibodies against Snail1, Sumo1, TβRI, Hes1 and c-Jun were used for staining. DAB chromogen (brown color) was used for the detection and the sections were counterstained with hematoxylin to visualize nuclei (blue). The stained tissues were dried overnight and then scanned with Panoramic 250 Flash scanner (3D HISTECH Ltd). Ethical permit to use tumor tissues for generation of tissue slides was provided by the Umeå University. Ethical review board in full agreement with the Swedish Ethical Review Act (540/03, Dnr 03-482).

### Statistical analysis of qRT-PCR data

After qRT-PCR, the cycle threshold (C_T_) values of respective genes were normalized to the C_T_ of the reference gene *GAPDH*. Then, the ∆ C_T_ values were obtained and plotted. We performed statistical analysis for each of three or more independent qRT-PCR assays. Bars are presented as means ± SEM from at least three independent experiments. Differences in the means ± SEM between samples were analyzed with two-way ANOVA with bonferroni post hoc test using SPSS software.

## SUPPLEMENTARY MATERIALS FIGURES


