# Supplementary material for: Pro-invasive properties of Snail1 are regulated by sumoylation in response to TGFβ stimulation in cancer
Source: Oncotarget. 2017 Aug 9;8(58):97703–26. doi: 10.18632/oncotarget.20097 (PMC5716685; doi:10.18632/oncotarget.20097)
Supplement: Supplementary file 1 [file oncotarget-08-97703-s001.pdf]

Pro-invasive properties of Snail1 are regulated by sumoylation in response to TGFβ stimulation in cancer

SUPPLEMENTARY MATERIALS

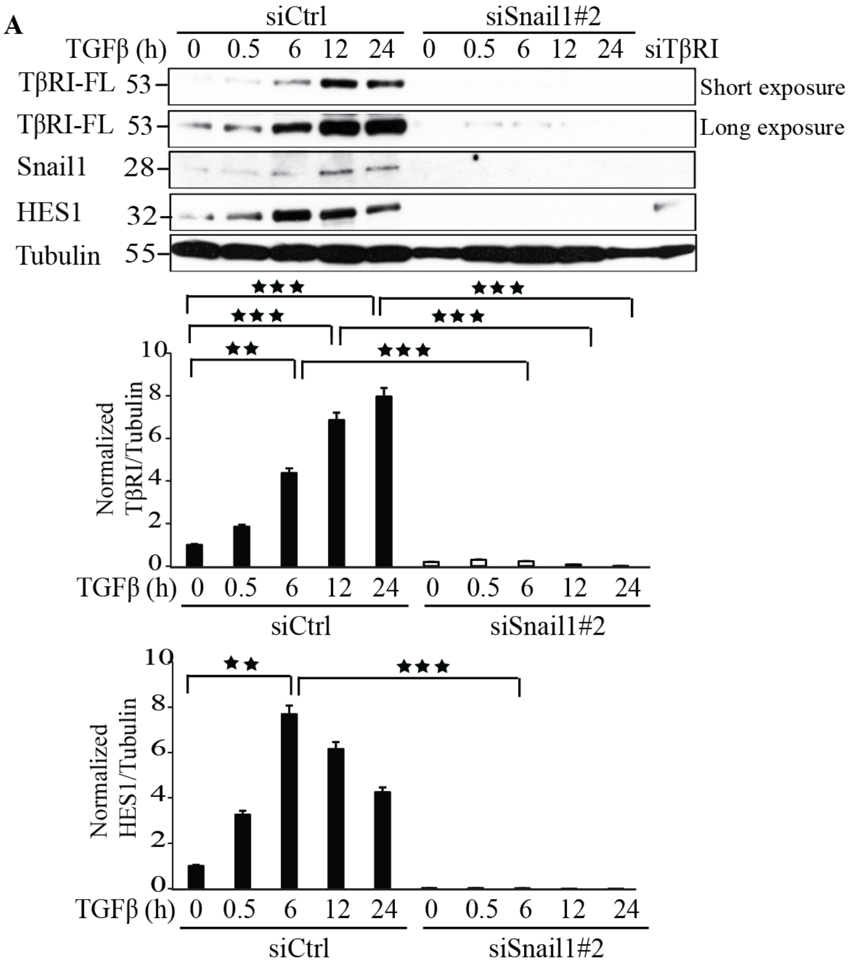

Supplementary Figure 1: Snail1 regulates TβRI and other EMT genes transcription. (**A**) PC-3U cells were transiently transfected with control (Ctrl) or Snail1-specific siRNA#2 treated with TGFβ (10 ng/ml) for the indicated time periods. Cell lysates were prepared and immunoblots were probed for TβRI (TβRI-FL), Snail1, Hes1, and β-tubulin, which served as control for equal loading of proteins. (n = 4 independent experiments). Bar graphs show the means ± SEM; $^{*}P< 0.05$, $^{**}P< 0.005$, $^{***}P< 0.0005$. Differences in the means ± SEM between samples were analyzed with two-way ANOVA and Bonferroni correction for multiple comparisons.

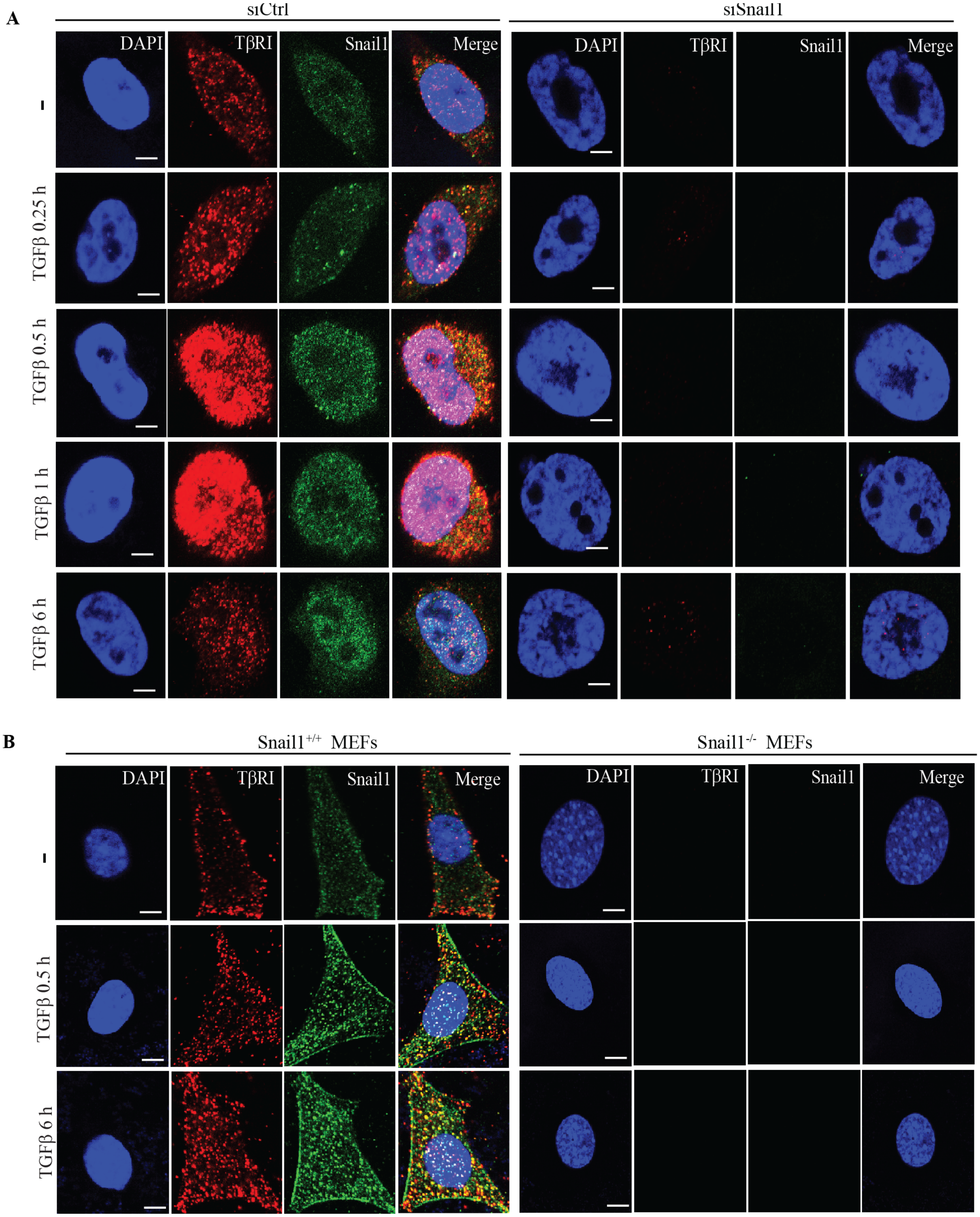

Supplementary Figure 2: Snail1 promotes expression of TβRI and co-localize with TβRI. (**A**) Confocal images of PC-3U cells transfected with control (Ctrl) or Snail1-specific siRNA #1. 48h post transfection, cells were incubated in low serum containing medium for 24 h followed by treatment with TGFβ (10 ng/ml) for the indicated time periods. Cells were fixed, permeabilised and incubated with anti-rabbit TβRI and anti-mouse Snail1 antibodies, followed by incubation with Alexa Fluor 555 (red) secondary anti-rabbit antibodies and Alexa Fluor 488 (green) secondary anti-mouse antibodies for visualization. Merge of two layers shows co-localization of the proteins. Cell nuclei stained with DAPI. (n=3 independent experiments). Scale bar, 20 µm; (**B**) Confocal images of Snail1$^{+/+}$ and Snail1$^{-/-}$ MEFs treated with TGFβ (10 ng/ml) for the indicated time periods. Cells were fixed, permeabilised and incubated with anti-rabbit TβRI and anti-mouse Snail1 antibodies, followed by incubation with Alexa Fluor 555 (red) secondary anti-rabbit antibodies and Alexa Fluor 488 (green) secondary anti-mouse antibodies for visualization. Merge of two layers shows co-localization of the proteins. Cell nuclei stained with DAPI (n=3 independent experiments). Scale bar, 20 µm.

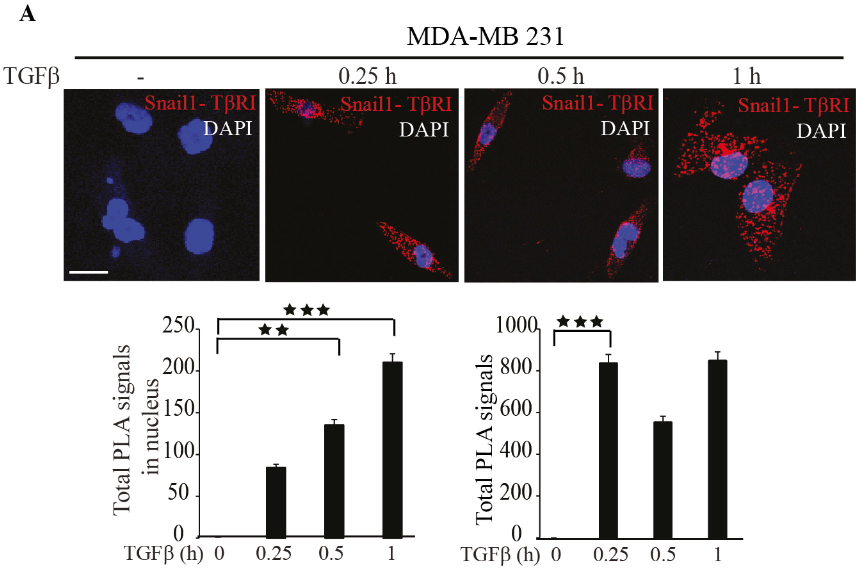

Supplementary Figure 3: TGFβ stimulates sumoylation of Snail1 in aggressive breast carcinoma MDA-MB-231 cells. (**A**) PLA images of MDA-MB-231 cells treated with TGFβ for the indicated time periods. Cells were fixed, permeabilised and incubated with anti-rabbit TβRI and anti-mouse Snail1 antibodies, followed by incubation with PLA probes. TβRI-Snail1 PLA complexes are visualized as red dots. Quantification of TβRI-Snail1 PLA complexes was done with the aid of Blob finder software. (n = 3 independent experiments). Bar graphs show the means ± SEM; $^{**}P< 0.005$, $^{***}P< 0.0005$. Differences in the means ± SEM between samples were analyzed with two-way ANOVA and Bonferroni correction for multiple comparisons.

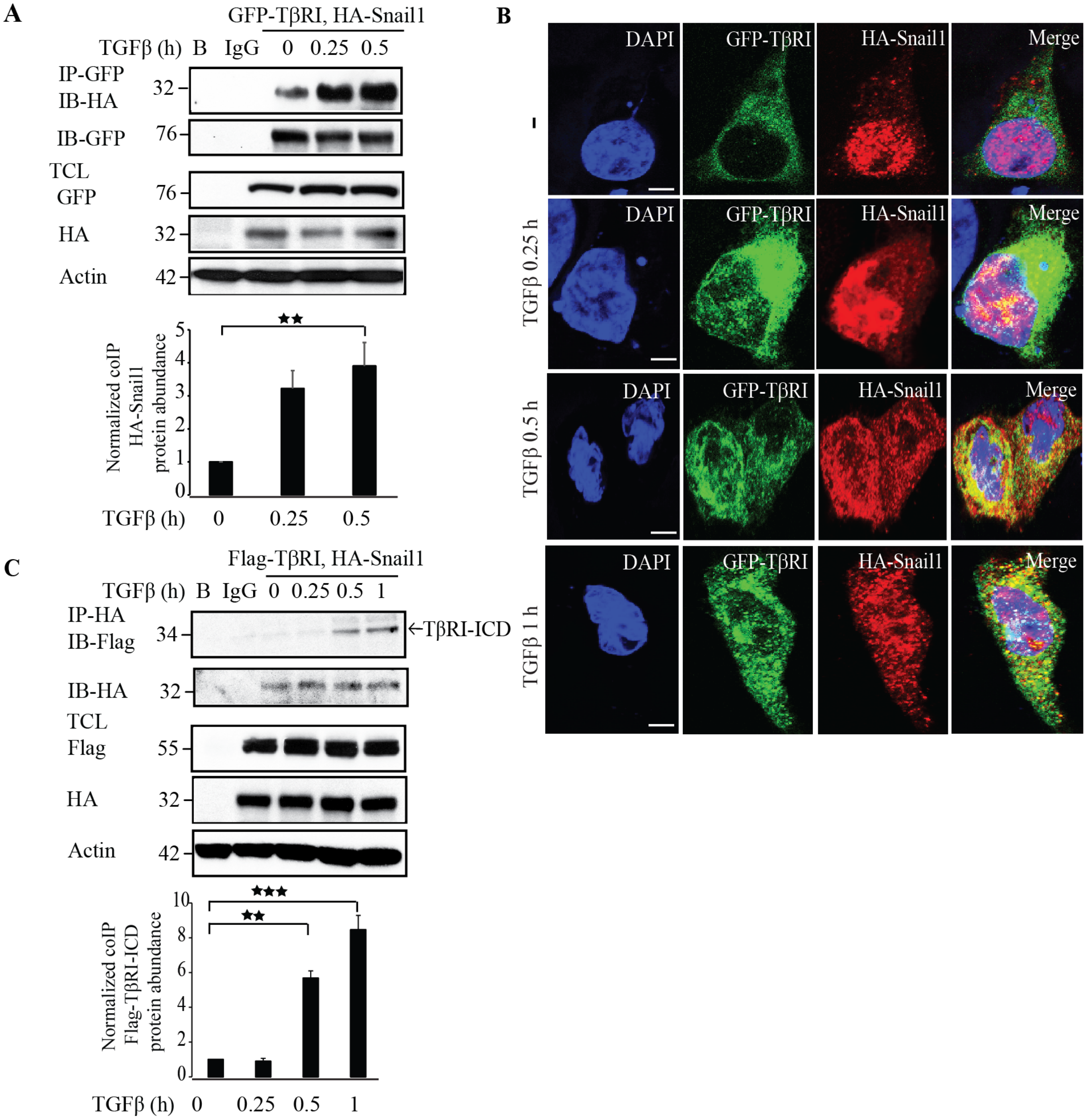

Supplementary Figure 4: TGFβ promotes complex formation of Snail and TβRI-ICD. (**A**) PC-3U cells were transiently co-transfected with GFP-TβRI (GFP-tagged towards c-terminus of TβRI) and HA-Snail1 and treated with TGFβ for the indicated time periods. TβRI was immunoprecipitated from cell lysates with GFP antibodies. Co-immunoprecipitated Snail1 and TβRI was detected by immunoblotting for HA and GFP. The levels of GFP-TβRI, HA-Snail1 and β-actin, was determined by immunoblotting of total cell lysates. (n=5 independent experiments). (**B**) Confocal images of ectopically expressed GFP-TβRI and HA-Snail1 in PC-3U cells, visualized with GFP (green) and HA (red) antibodies. Cells were fixed, permeabilised and incubated with anti-rabbit HA and anti-mouse GFP antibodies, followed by incubation with Alexa Fluor 555 (red) secondary anti-rabbit antibodies and Alexa Fluor 488 (green) secondary anti-mouse antibodies for visualization. Merge of two layers shows co-localization of the proteins. Cell nuclei stained with DAPI. (n=3 independent experiments). Scale bar, 20 µm. (**C**) PC-3U cells were transiently co-transfected with Flag-TβRI (Flag-tagged towards c-terminus of TβRI) and HA-Snail1 and treated with TGFβ for the indicated time periods. TβRI was immunoprecipitated from cell lysates with HA antibodies. Coimmunoprecipitated Snail1 and TβRI was detected by immunoblotting for Flag and HA. The levels of Flag-TβRI and HA-Snail1 and β-actin were determined by immunoblotting of total cell lysates. (n=3 independent experiments). Bar graphs show the means ± SEM; $^{**}P < 0.005$, $^{***}P < 0.0005$. Differences in the means ± SEM between samples were analyzed with two-way ANOVA and Bonferroni correction for multiple comparisons.

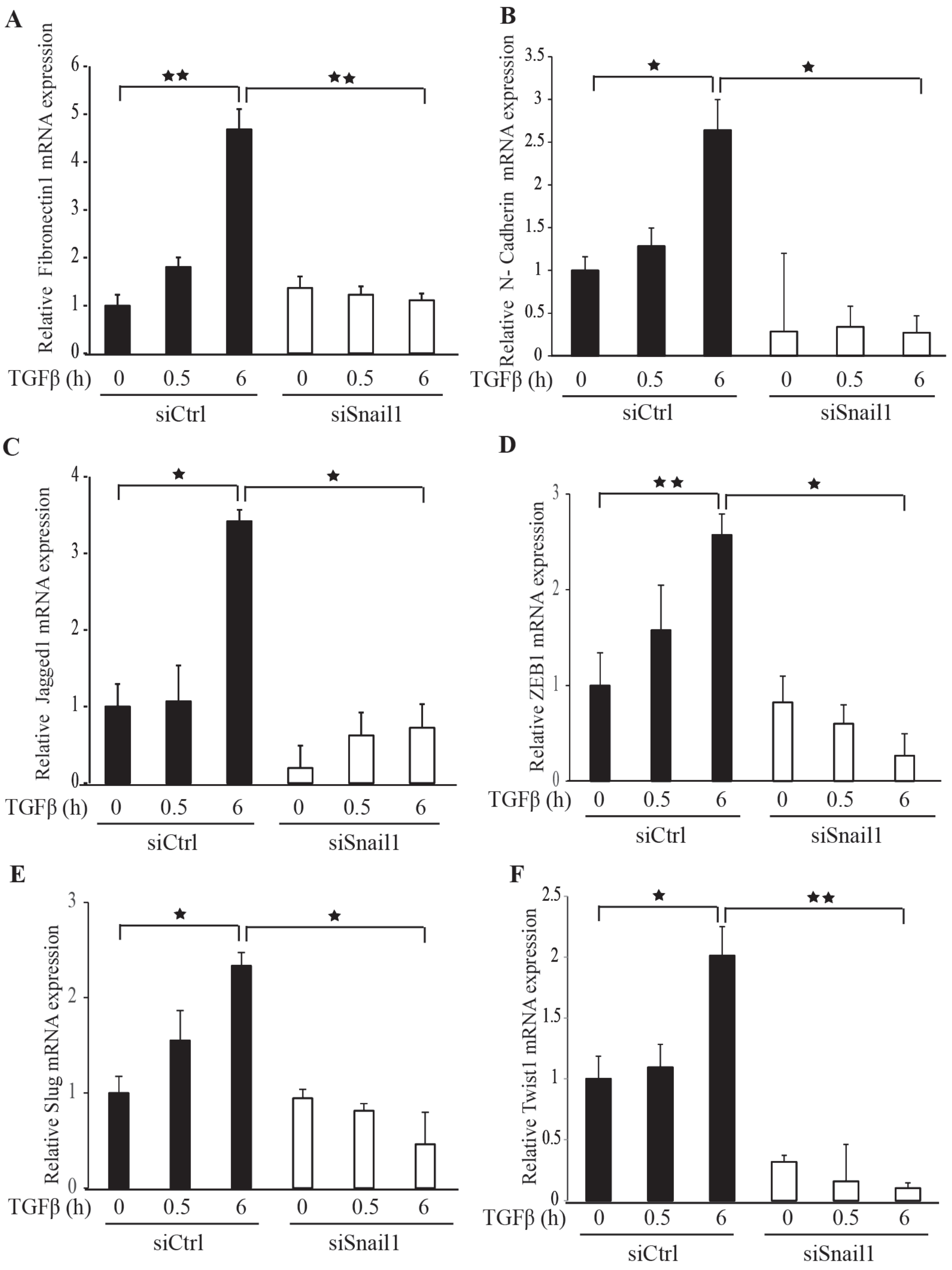

Supplementary Figure 5: Snail1 regulates transcription of EMT genes. qRT-PCR analysis of PC-3U cells transiently transfected with control (Ctrl) or Snail1-specific siRNA #1 and treated with TGFβ (10 ng/ml) as indicated. RNA was extracted and cDNA was prepared and used for qRT-PCR analysis of mRNA expression of mesenchymal markers Fibronectin1, N-cadherin, the notch responsive gene Jagged1 (Supplementary 5A-C) and other EMT regulators such as Zeb1, Slug, and Twist1 (Supplementary 5D-F). Bar graphs show the means ± SEM; $^{*}P< 0.05$, $^{**}P< 0.005$, $^{***}P< 0.0005$. Differences in the means ± SEM between samples were analyzed with two-way ANOVA and Bonferroni correction for multiple comparisons.

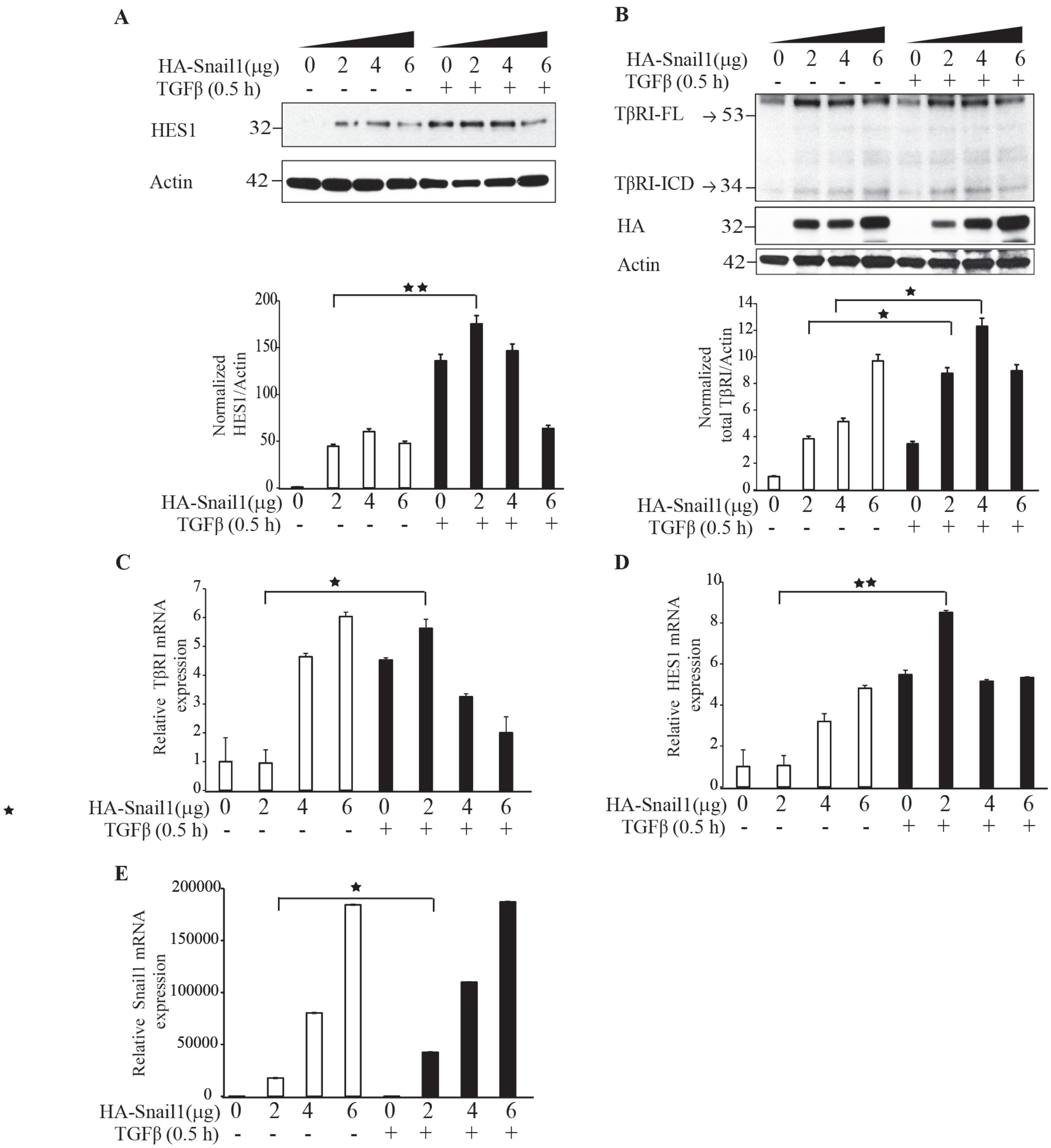

Supplementary Figure 6: Snail1 overexpression enhances TβRI-ICD generation and Hes1 expression. (**A**, **B**) PC-3U cells were transiently transfected with increasing concentrations of HA-Snail1, stimulated with TGFβ for the indicated time periods. Representative immunoblots of total cell lysates probed with antibodies specific to Hes1, TβRI, HA, and β-actin. (n=4 independent experiments). (**C**, **D**, **E**) qRT-PCR analysis of PC-3U cells transiently transfected with increasing concentrations of HA-Snail1, stimulated with TGFβ for the indicated time periods. RNA was extracted and cDNA was prepared and used for qRT-PCR analysis of mRNA expression of TβRI, Hes1, and Snail1. (n=5 independent experiments). Bar graphs show the means ± SEM; $^{*}P < 0.05$, $^{**}P < 0.005$. Differences in the means ± SEM between samples were analyzed with two-way ANOVA and Bonferroni correction for multiple comparisons.

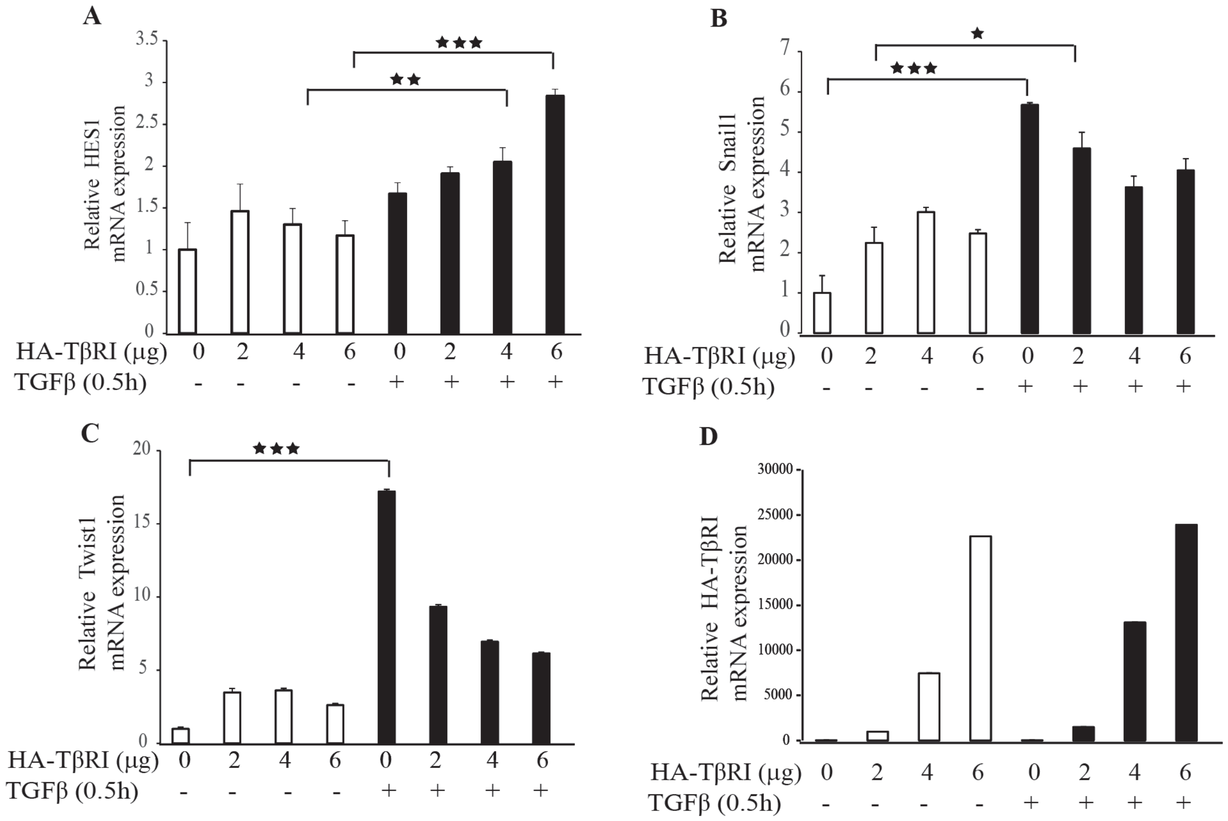

Supplementary Figure 7: TβRI overexpression enhances Hes1 expression. (**A-D**) qRT-PCR analysis of PC-3U cells transiently transfected with increasing concentrations of HA-TβRI, stimulated with TGFβ for the indicated time periods. RNA was extracted and cDNA was prepared and used for qRT-PCR analysis of mRNA expression of Hes1, Snail1, Twist1 and TβRI. (n=4 independent experiments). Bar graphs show the means ± SEM; $^{*}P< 0.05$, $^{**}P< 0.005$, $^{***}P< 0.0005$. Differences in the means ± SEM between samples were analyzed with two-way ANOVA and Bonferroni correction for multiple comparisons.

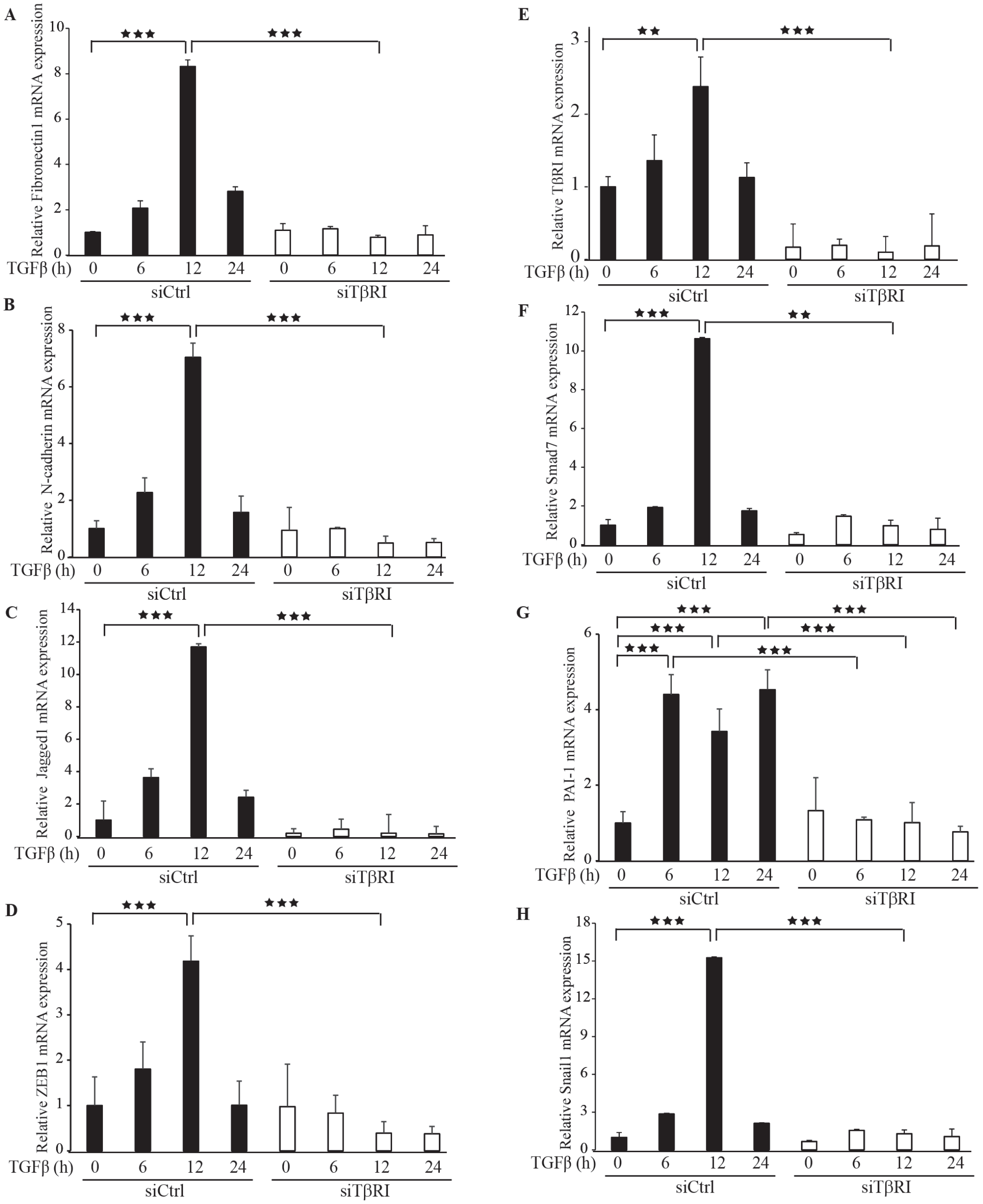

Supplementary Figure 8: TβRI regulates EMT genes. (**A-H**) qRT-PCR analysis of PC-3U cells transiently transfected with control (Ctrl) or TβRI -specific siRNA and treated with TGFβ (10 ng/ml) as indicated. RNA was extracted and cDNA was prepared and used for qRT-PCR analysis of mRNA expression of mesenchymal markers Fibronectin1, N-cadherin, the notch responsive gene Jagged1, Zeb1, TβRI, TGFβ target genes Smad7, PAI-1, and Snail1. (n=5 independent experiments). Bar graphs show the means ± SEM; $^{**}P< 0.005$, $^{***}P< 0.0005$. Differences in the means ± SEM between samples were analyzed with two-way ANOVA and Bonferroni correction for multiple comparisons.

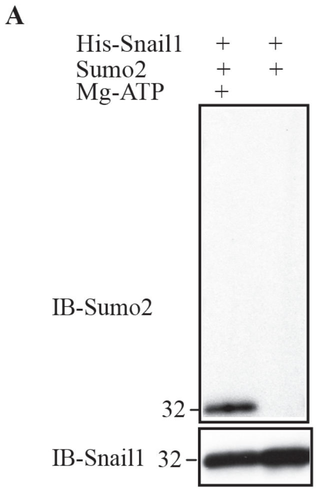

Supplementary Figure 9: Snail1 sumoylation is not dependent on SUMO2. (**A**) His-Snail1 was incubated with SUMO2 and subjected to *In vitro* sumoylation reaction for 60 min in the presence or absence of Mg-ATP. Reaction was terminated by adding sample buffer and boing at 95°C. Samples were immunoblotted with anti-SUMO2 and anti-Snail1 antibodies. (n=4 independent experiments).

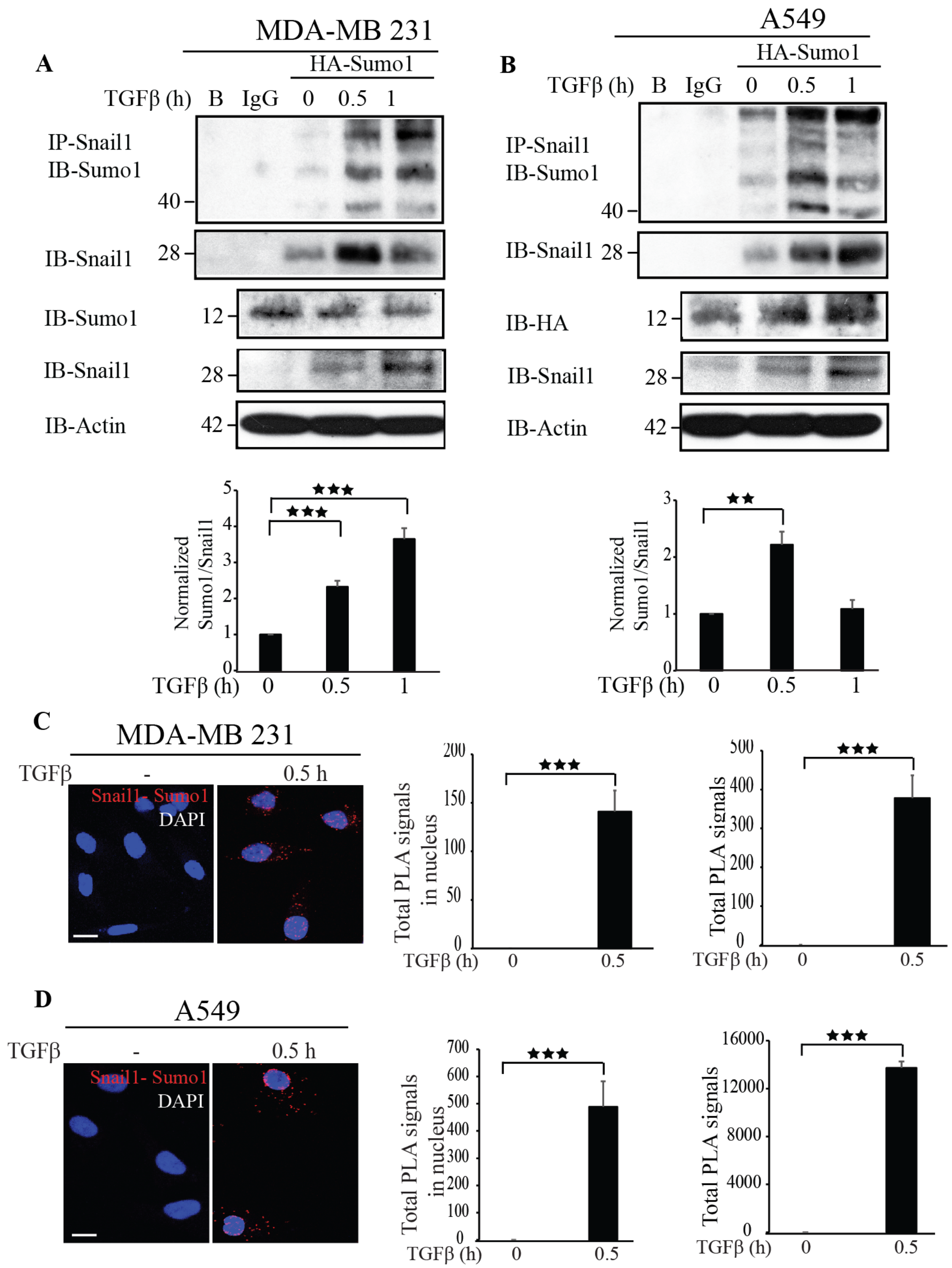

Supplementary Figure 10: TGFβ stimulates sumoylation of Snail1 in MDA-MB-231 or A549 cells. (**A**, **B**) MDA-MB-231 cells or A549 cells were transiently transfected with HA-Sumo1 and treated with TGFβ for the indicated time periods. Cell lysates were subjected to sumoylation assay and immunoprecipitated with goat-Snail1 and immunoblotted with rabbit-Sumo1 antibodies. Total cell lysates were probed for Sumo1, Snail1, and β-actin. (n=4 independent experiments). (**C**, **D**) PLA images of PC-3U cells were treated with TGFβ for the indicated time periods. Cells were fixed, permeabilised and incubated with anti-rabbit Sumo1 and anti-mouse Snail1 antibodies, followed by incubation with PLA probes. Snail1-Sumo1 PLA complexes are visualized as red dots. Quantification of Snail1-Sumo1 PLA complexes was done with the aid of Blob finder software. (n = 3 independent experiments). Bar graphs show the means ± SEM; $^{**}P < 0.005$, $^{***}P < 0.0005$. Differences in the means ± SEM between samples were analyzed with two-way ANOVA and Bonferroni correction for multiple comparisons.

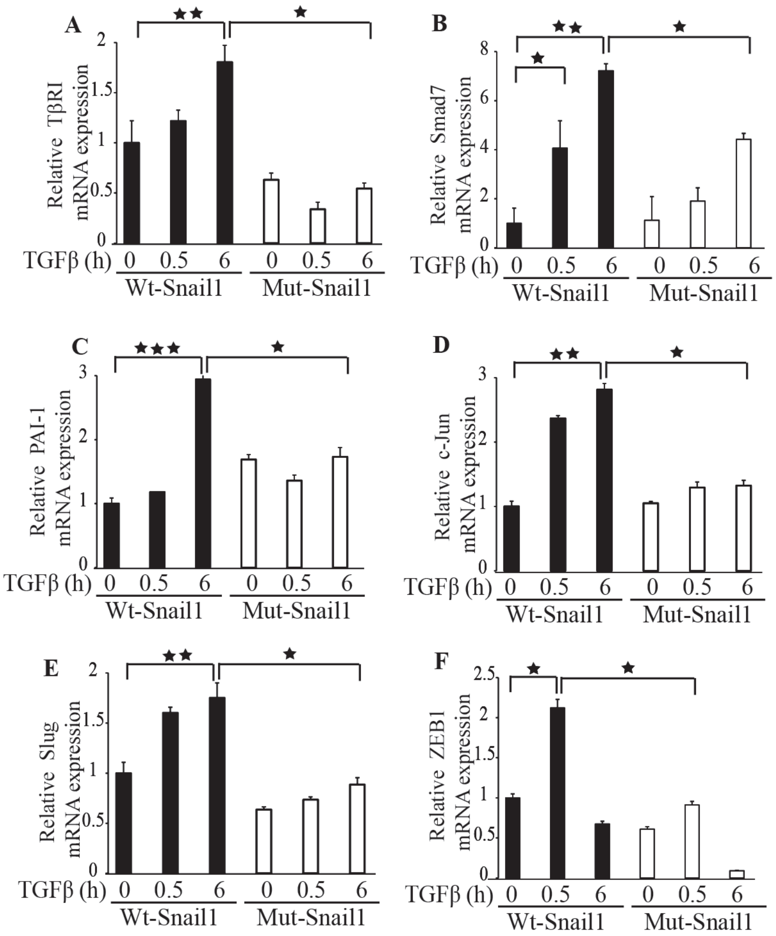

Supplementary Figure 11: SnailK234R mutant can't promote TGFβ-regulated EMT target gene responses. qRT-PCR analysis of PC-3U cells transiently transfected with HA-WT-Snail1 or Mutant-HA-Snail1 and treated with TGFβ (10 ng/ml) for the indicated time periods. RNA was extracted and cDNA was prepared and used for qRT-PCR analysis of mRNA expression of TβRI, TGFβ target genes Smad7, PAI-1, c-Jun and EMT regulators Slug, Zeb1, (n=5 independent experiments). Bar graphs show the means ± SEM; $^{*}P< 0.05$, $^{**}P< 0.005$, $^{***}P< 0.0005$. Differences in the means ± SEM between samples were analyzed with two-way ANOVA and Bonferroni correction for multiple comparisons.

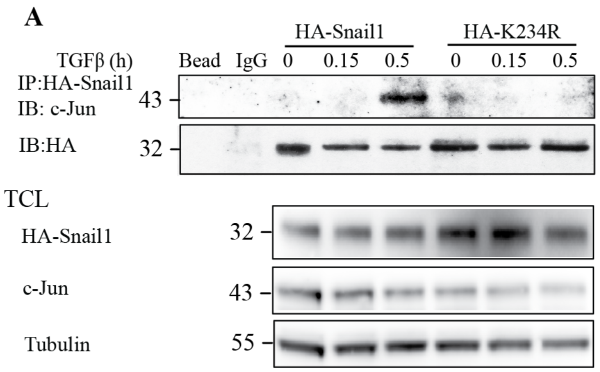

Supplementary Figure 12: Wild type Snail1 interacts with c-Jun. (**A**) PC-3U cells were transiently transfected with wild type HA-Snail1 or mutant HA-Snail1 (K234R) and treated with TGFβ for the indicated time periods. Snail1 was immunoprecipitated from cell lysates with HA antibodies. Co-immunoprecipitated c-Jun and HA-Snail1, was detected by immunoblotting for HA and c-Jun. The levels of HA-Snail1, c-Jun and β-actin was determined by immunoblotting of total cell lysates. (n = 4 independent experiments).

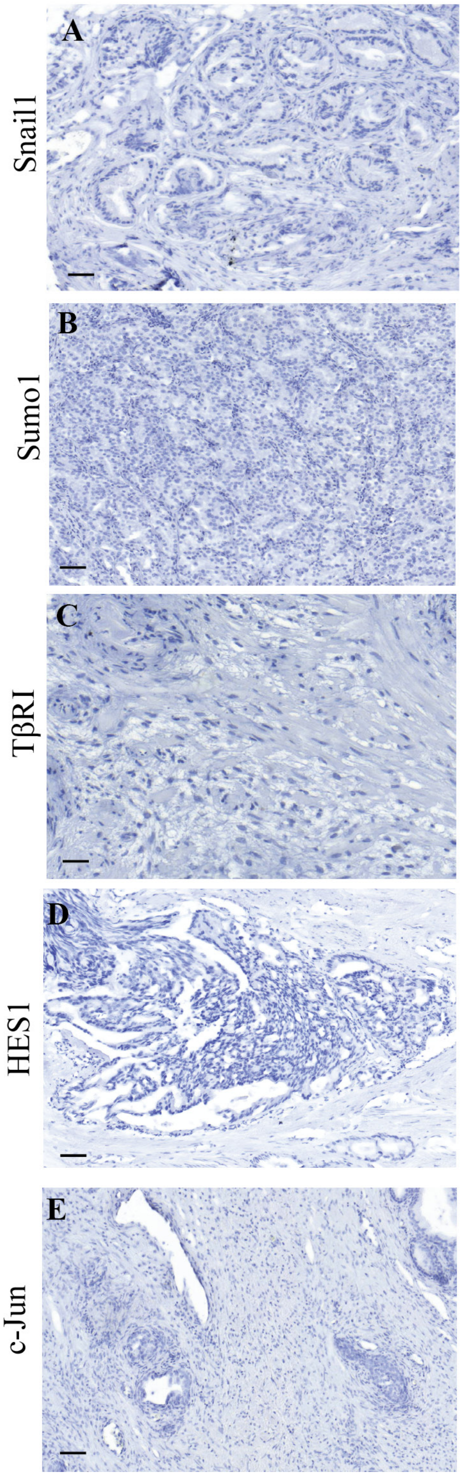

Supplementary Figure 13: Control (-ve) immunohistochemical staining's of prostate cancer tissues. (**A**-**E**) Paraffin-embedded sections of prostate cancer tissues were the primary antibody was omitted and immunostained was used as a negative control. Representative images from cancer tissues (Scale bar 200 µm).
